# Targeting CCR5 with miltefosine as a therapeutic strategy for thrombocytopenia

**DOI:** 10.1016/j.isci.2025.112379

**Published:** 2025-04-08

**Authors:** Qinyao Li, Ting Zhang, Zhichao Li, Xiao Qi, Xinyue Mei, Sheng Liu, Siyu He, Gan Qiao, Rong Li, Hongping Shen, Jing Zeng, Feihong Huang, Shuang Dai, Sirui Li, Jiesi Luo, Jianming Wu, Long Wang

**Affiliations:** 1 Department of Pharmacology, School of Pharmacy, Southwest Medical University, Luzhou, Sichuan 646000, China; 2 School of Basic Medical Sciences, Southwest Medical University, Luzhou, Sichuan 646000, China; 3 Department of Pharmacy, Longquanyi District of Chengdu Maternity & Child Health Care Hospital, Chengdu, Sichuan 610100, China; 4 Drug Discovery Research Center, Southwest Medical University, Luzhou, Sichuan 646000, China; 5 Laboratory for Cardiovascular Pharmacology of Department of Pharmacology, The School of Pharmacy, Southwest Medical University, Luzhou, Sichuan 646000, China; 6 Clinical Trial Center, The Affiliated Traditional Chinese Medicine Hospital of Southwest Medical University, Luzhou, Sichuan 646000, China; 7 Education Ministry Key Laboratory of Medical Electrophysiology, Sichuan Key Medical Laboratory of New Drug Discovery and Druggability Evaluation, Luzhou Key Laboratory of Activity Screening and Druggability Evaluation for Chinese Materia Medica, Southwest Medical University, Luzhou, Sichuan 646000, China

**Keywords:** Molecular biology, Immunology

## Abstract

Thrombocytopenia remains a challenging clinical condition with limited treatment options. Here, we demonstrated that miltefosine stimulated megakaryocyte (MK) differentiation *in vitro*. Miltefosine significantly accelerated platelet recovery, enhanced platelet function, and boosted MK production and differentiation in irradiated mice. RNA sequencing revealed association of CCR5, MAPK, and JAK2/STAT3 signaling pathways in miltefosine-mediated MK differentiation. Molecular docking, drug affinity responsive target stability (DARTS), and surface plasmon resonance (SPR) assays confirmed direct binding of miltefosine to CCR5. Inhibition of CCR5 disrupted miltefosine’s effects on MK differentiation and activation of MAPK and JAK2/STAT3 signaling pathways, as well as key transcription factors GATA1, EGR1, and TAL1. Similarly, blockade of the MAPK or JAK2/STAT3 signaling pathways hindered miltefosine-induced MK differentiation and transcription factor activation. Our findings establish CCR5 as a therapeutic target for thrombocytopenia and identify miltefosine as a CCR5 agonist that promotes MK differentiation and platelet production via MAPK and JAK2/STAT3 signaling.

## Highlights

• Miltefosine stimulates megakaryocyte differentiation
• Miltefosine accelerates platelet recovery in irradiated mice
• Miltefosine binds directly to CCR5, activating MAPK and JAK2/STAT3 pathways
• Miltefosine represents a promising therapeutic approach for thrombocytopenia

## Graphical abstract

**Figure:**
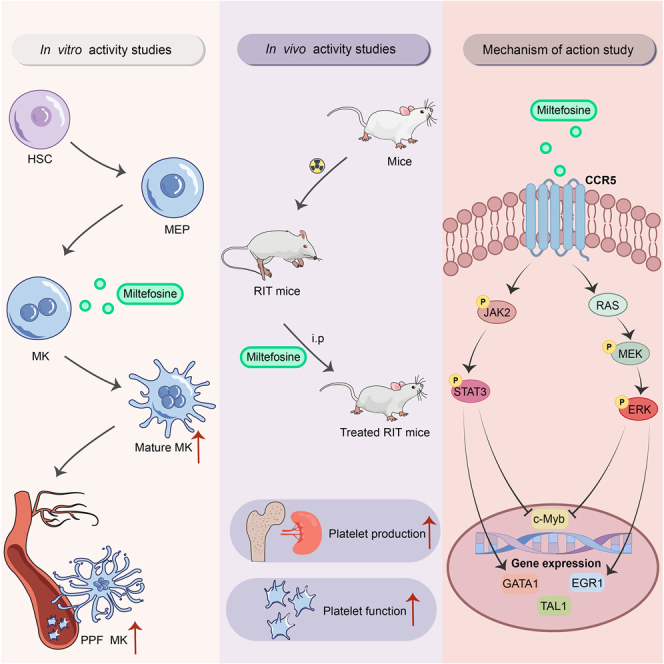

## Introduction

Platelets, anucleated cell fragments, are indispensable components of the circulatory system. Beyond their well-established role in hemostasis, platelets are increasingly recognized for their functions in physiological processes, including vascular repair, inflammation, immune responses, and tumorigenesis.^1^^,^^2^ Thrombopoiesis, the process of platelet generation, initiates within the bone marrow (BM) from hematopoietic stem cells (HSCs). HSCs embark on a differentiation pathway, culminating in the production of platelets. These pluripotent progenitors first differentiate into common myeloid progenitors (CMPs), which subsequently give rise to megakaryocyte-erythroid progenitors (MEPs). MEPs represent a critical bifurcation point, with the potential to develop into either megakaryocytes (MKs) or erythrocytes. Committed MK progenitors emerge from MEPs and undergo a series of differentiation and maturation steps. A hallmark of MK development is endomitosis, a unique process characterized by multiple rounds of DNA replication without cytokinesis, leading to polyploid giant cells. This cellular enlargement provides the requisite platform for subsequent platelet biogenesis. Mature MKs extend proplatelet processes into the BM sinusoids, where shear forces exerted by blood flow induce their fragmentation into nascent platelets. Each MK is capable of producing thousands of platelets, highlighting the efficiency of this hematopoietic process.^1^^,^^3^^,^^4^

MK differentiation and maturation is a complex process regulated by cytokines, hormones, transcription factors, non-coding RNAs, and metabolites.^4^^,^^5^ Thrombopoietin (TPO) serves as a pivotal regulator, binding to c-Mpl to activate JAK2/STATs, PI3K/Akt, and MAPK signaling pathways. These signaling cascades subsequently activate hematopoietic transcription factors such as GATA1, TAL1, and EGR1, driving MK differentiation and maturation.^6^ Beyond TPO, chemokines like C-C motif chemokine ligand 5 (CCL5, RANTES), upon binding to CC chemokine receptor 5 (CCR5), activate the Akt pathway, promoting MK maturation and proplatelet formation.^7^ This intricate signaling network orchestrates the precise regulation of thrombopoiesis.

Thrombocytopenia, characterized by a platelet level below 150×10^9^/L, is a common hematological disorder that can result from impaired platelet production, increased destruction, or dysfunctional platelets.^8^ Diverse etiologies, such as BM disorders, autoimmune diseases, and drug-induced adverse effects, contribute to occurrence of thrombocytopenia.^8^^,^^9^ Individuals with thrombocytopenia face a heightened risk of bleeding complications, ranging from mucocutaneous bleeding to life-threatening hemorrhage.^8^ Radiation therapy is a mainstay in cancer treatment. However, its efficacy in eradicating tumor cells is often accompanied by significant BM suppression, leading to radiation-induced thrombocytopenia (RIT). This complication not only limits the dose and duration of radiation therapy but also predisposes patients to increased risks of infection and bleeding, thereby compromising patient quality of life and overall prognosis.^9^^,^^10^ Recombinant human thrombopoietin (rhTPO) and thrombopoietin receptor agonists (TPO-RAs), including eltrombopag, avatrombopag, and romiplostim, are currently the mainstay of therapy for thrombocytopenia. These agents stimulate megakaryopoiesis and effectively elevate platelet counts, reducing the need for platelet transfusions.^11^^,^^12^ However, their clinical utility is limited by several factors. Excessive platelet production can lead to a heightened risk of thrombosis, while off-target effects such as myelofibrosis, immune-related adverse events (including infections and autoimmune disorders), and hepatic injury are also commonly observed.^11^^,^^12^ As a result, it is crucial to develop innovative, effective, and safe thrombopoietic drugs to meet the existing clinical demands.

Miltefosine, an alkylphosphocholine compound, exhibits broad-spectrum antiprotozoal activity.^13^ Initially explored for its anticancer properties, the drug subsequently demonstrated potent efficacy against leishmania species, positioning it as a front line oral therapy for leishmaniasis.^13^ Intriguingly, a clinical trial involving 72 cancer patients demonstrated a marked elevation in platelet level (1.19– to2.33-fold) in 73% of participants following miltefosine administration.^14^ This observation, coupled with the absence of myelosuppression in preclinical studies at therapeutic doses,^14^^,^^15^^,^^16^ suggests a potential role for miltefosine in promoting thrombopoiesis. Elucidating the precise mechanisms underlying this effect warrants further investigation.

To explore the therapeutic potential of miltefosine in thrombocytopenia, we first characterized its effects on MK differentiation *in vitro*. We subsequently evaluated its efficacy in treating RIT *in vivo*. Mechanistically, RNA sequencing and validation experiments revealed that miltefosine functions as a CCR5 agonist, stimulating MK differentiation by regulation of JAK2/STAT3 and MEK/ERK signaling pathways. Our findings establish miltefosine as a CCR5 agonist with therapeutic promise for thrombocytopenia.

## Results

### Miltefosine enhances MK differentiation of K562 and HEL cells

**Figure 1:**
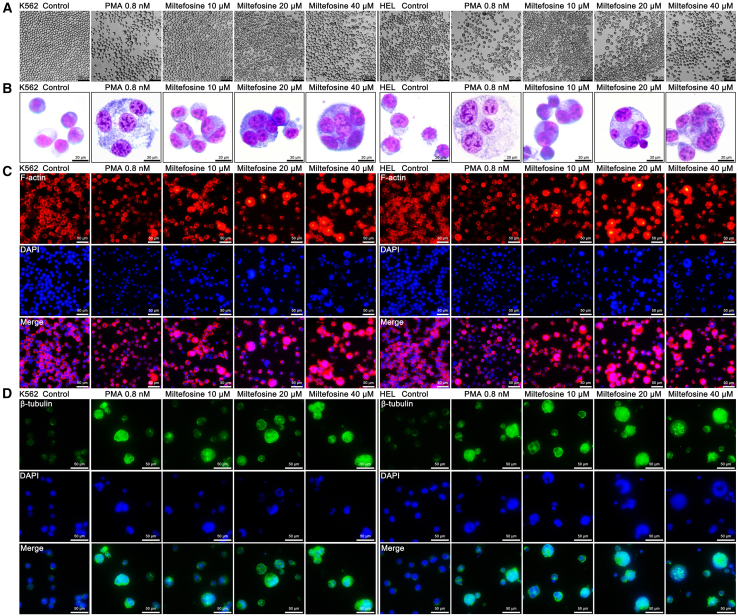
Miltefosine induces morphological changes in K562 and HEL cells (A) Representative bright-field images of K562 and HEL cells treated with miltefosine (10, 20, and 40 μM) or PMA (0.8 nM) for 5 days. Scale bar: 100 μM. (B) Giemsa staining of K562 and HEL cells after 5 days of treatment with miltefosine (10, 20, and 40 μM) or PMA (0.8 nM). Scale bar: 20 μM. (C) Phalloidin staining of F-actin in K562 and HEL cells treated with miltefosine (10, 20, and 40 μM) or PMA (0.8 nM) for 5 days. Scale bar: 50 μM. (D) Immunofluorescence staining of β-tubulin in K562 and HEL cells treated with miltefosine (10, 20, and 40 μM) or PMA (0.8 nM) for 5 days. Scale bar: 50 μM.

**Figure 2:**
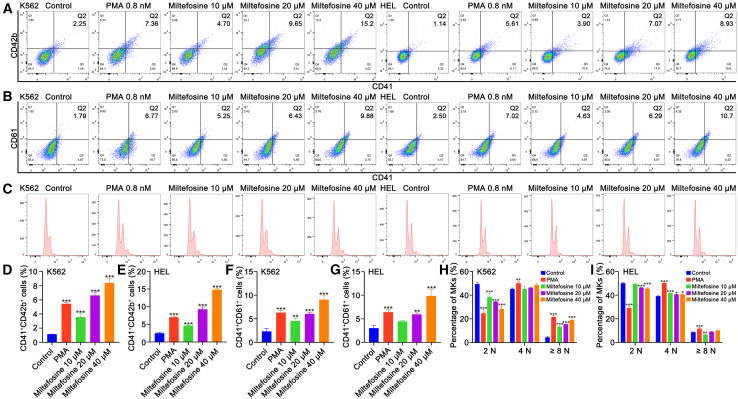
Miltefosine promotes MK differentiation and maturation in K562 and HEL cells (A) Flow cytometric analyses of proportion of CD41^+^CD42b^+^ cells in K562 and HEL cells treated with miltefosine (10, 20, and 40 μM) or PMA (0.8 nM) for 5 days. (B) Flow cytometric analyses of proportion of CD41^+^CD61^+^ cells of each group. (C) DNA ploidy of each group. (D) Quantification of the percentage of CD41^+^CD42b^+^ cells in K562. *n* = 3. (E) Quantification of the percentage of CD41^+^CD42b^+^ cells in HEL. *n* = 3. (F) Quantification of the percentage of CD41^+^CD61^+^ cells in K562. *n* = 3. (G) Quantification of the percentage of CD41^+^CD61^+^ cells in HEL. *n* = 3. (H) Quantification of the DNA ploidy in K562. *n* = 3. (I) Quantification of the DNA ploidy in HEL. *n* = 3. Statistical significance in (D–I) was calculated using a one-way analysis of variance (ANOVA) test. ∗*p* < 0.05, ∗∗*p* < 0.01, ∗∗∗*p* < 0.001, vs. control group. Error bars represent mean ± SD.

To investigate the potential of miltefosine to promote MK differentiation, we utilized K562 and HEL cells. The effect of miltefosine on cell proliferation was first detected. Our data demonstrated a dose-dependent inhibition of cell proliferation by miltefosine (2.5–320 μM) (Figures S1A and S1B), suggesting that miltefosine induces a cellular environment conducive to differentiation. We subsequently treated K562 and HEL cells with miltefosine (10, 20, and 40 μM) and compared the effects to those induced by PMA (0.8 nM), a known inducer of MK differentiation. Notably, both miltefosine and PMA treatments resulted in the emergence of large, morphologically distinct cells, a phenotype that was not observed in the control group (Figure 1A). Giemsa staining showed an increase in multinucleated cells in both miltefosine and PMA-treated groups, indicating enhanced nuclear maturation (Figure 1B). Further, phalloidin staining confirmed the presence of multilobulated nuclei in treated cells, contrasting with the relatively simpler nuclear morphology seen in control (Figure 1C). Given that tubulin rearrangement is a hallmark of MK terminal differentiation, we performed β-tubulin immunofluorescence staining. Both miltefosine and PMA significantly upregulated and aggregated β-tubulin expression (Figure 1D), consistent with cytoskeletal reorganization during differentiation. Flow cytometric analyses of CD41, CD42b, and CD61 surface markers, indicative of MK differentiation and maturation, revealed a marked increase in CD41^+^CD42b^+^ and CD41^+^CD61^+^ populations following treatment with miltefosine and PMA. This effect was dose-dependent in the case of miltefosine (Figures 2A, 2B, and 2D–2G). Additionally, analysis of DNA ploidy demonstrated a decrease in the 2 N cell population alongside an increase in cells with ≥8 N DNA content in K562 cells treated with miltefosine (Figures 2C and 2H). In HEL cells, we observed a reduction in 2 N cells and a corresponding rise in 4 N cells with increasing doses of miltefosine (Figures 2C and 2I). The results suggest that miltefosine promotes endomitosis, a key feature of MK maturation. Moreover, the effects of miltefosine on mitochondria were investigated. The analysis of mitochondrial membrane potential (MMP) revealed that miltefosine (10, 20, and 40 μM) enhanced MMP in a dose-dependent manner in K562 and HEL cells (Figures S2A–S2D). Miltefosine treatment also resulted in a dose-dependent increase in mitochondrial mass in K562 and HEL cells (Figures S2E–S2H). The results suggest that miltefosine’s ability to increase MMP and mitochondrial mass likely provides the necessary energy support for MK differentiation. Collectively, our results demonstrate that miltefosine facilitates MK differentiation and maturation *in vitro*.

### Miltefosine promotes platelet recovery and function without systemic toxicity in RIT mice

**Figure 3:**
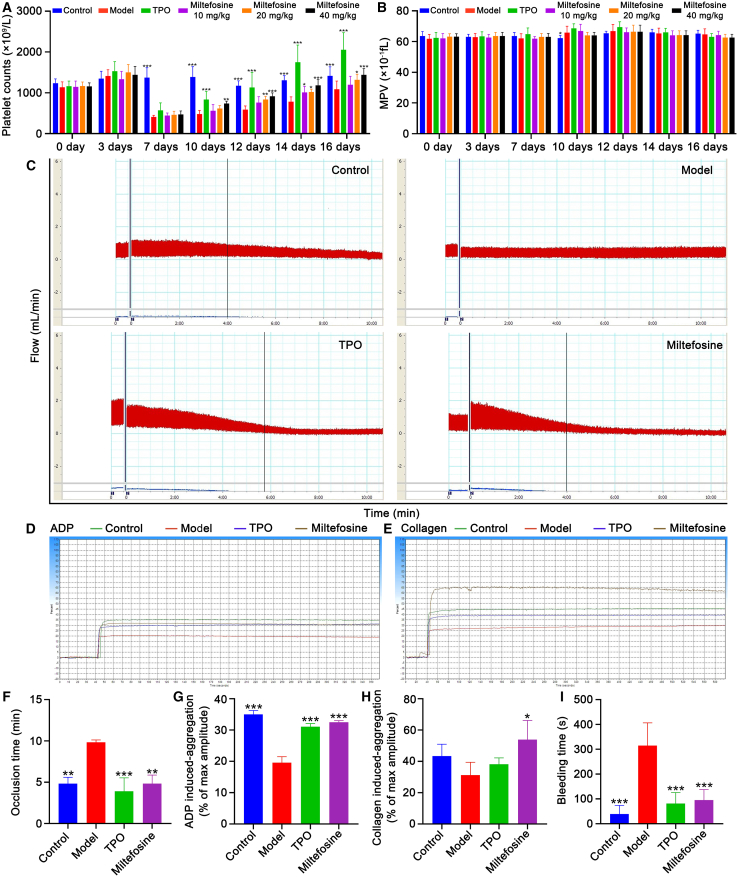
Miltefosine accelerates platelet recovery and improves function in RIT mice (A) Platelet counts in peripheral blood from day 0–16 in control, model, TPO (3000 U/kg), and miltefosine (10, 20, and 40 mg/kg)-treated groups. *n* = 10. (B) MPV in each group. *n* = 10. (C) Carotid blood flow tracings of each group. (D) Platelet aggregation induced by ADP stimulation in each group. (E) Platelet aggregation induced by collagen stimulation in each group. (F) Quantification of mean carotid artery occlusion times in each group. *n* = 3. (G) Quantification of maximum aggregation amplitude of platelets induced by ADP in each group. *n* = 3. (H) Quantification of maximum aggregation amplitude of platelets induced by collagen in each group. *n* = 3. (I) Quantification of tail bleeding time in each group. *n* = 3. Statistical significance in (A-B) and (F–I) was assessed using two-way ANOVA and one-way ANOVA tests, respectively. ∗*p* < 0.05, ∗∗*p* < 0.01, ∗∗∗*p* < 0.001, vs. model group. Error bars represent mean ± SD.

To assess the therapeutic potential of miltefosine for thrombocytopenia, we employed an RIT mouse model. Following 16 days of treatment, we observed that platelet counts in irradiated mice reached their nadir on day 7 and subsequently began to recover (Figure 3A). TPO-treated mice exhibited a more rapid platelet recovery compared to the model group, but with platelet counts exceeding normal levels on days 14 and 16 (Figure 3A), suggesting a potential thrombosis risk. In contrast, miltefosine-treated groups displayed a more measured recovery, with platelet counts increasing at a controlled rate without surpassing normal levels (Figure 3A), indicating a safer profile that minimizes the risk of excessive thrombosis. Importantly, mean platelet volume (MPV) remained unaffected by miltefosine throughout the treatment period (Figure 3B).

To evaluate the systemic safety of miltefosine, organ indices were detected and revealed no obvious changes in liver, spleen, lungs, kidneys, and thymus across all treatment doses (Figures S3A–S3E). Serum biochemical analyses revealed miltefosine did not affect alanine aminotransferase (ALT), aspartate aminotransferase (AST), or creatinine (CREA) contents, and low-dose miltefosine (10 mg/kg) notably reduced the elevated blood urea nitrogen (BUN) levels caused by irradiation (Figures S3F–S3I). H&E staining showed no significant changes in the structure of the heart, liver and kidney (Figure S3J).These findings indicate that miltefosine does not induce systemic toxicity in RIT mice.

Further analysis of platelet function demonstrated that miltefosine-treated mice, similar to the TPO-treated group, had significantly shorter occlusion times in the FeCl_3_-induced carotid artery thrombosis test compared to the model group, suggesting enhanced thrombosis formation upon vascular injury (Figures 3C and 3F). Additionally, platelet aggregation assays revealed that both adenosine diphosphate (ADP) and collagen-induced aggregation were stronger in miltefosine-treated group compared to model group (Figures 3D, 3E, 3G, and 3H). A tail bleeding assay corroborated these results, showing a significantly reduced bleeding time in miltefosine-treated group relative to model group, indicating enhanced coagulation function (Figure 3I). To assess whether miltefosine activates platelets, flow cytometry was used to analyze the expression of P-selectin (CD62P), a specific marker for platelet activation.^9^ The results showed that the expression of CD62P in platelets from the model group was significantly higher than in the control, TPO, and miltefosine-treated groups (Figures S4A and S4B), indicating that irradiation led to an increased expression of CD62P and possibly abnormal platelet activation. Under ADP stimulation, the model group exhibited a significantly reduced responsiveness to ADP, with lower CD62P expression, whereas the control, TPO, and miltefosine-treated groups showed a more sensitive response to ADP, with significantly higher CD62P expression than the model group (Figures S4C and S4D). These results suggest that miltefosine can activate platelets, which may help them perform their hemostatic function and prevent abnormal bleeding induced by thrombocytopenia. Collectively, these data suggest that miltefosine effectively enhances platelet recovery and function in RIT mice without causing systemic toxicity.

### Miltefosine rescues MK production and differentiation in RIT mice

**Figure 4:**
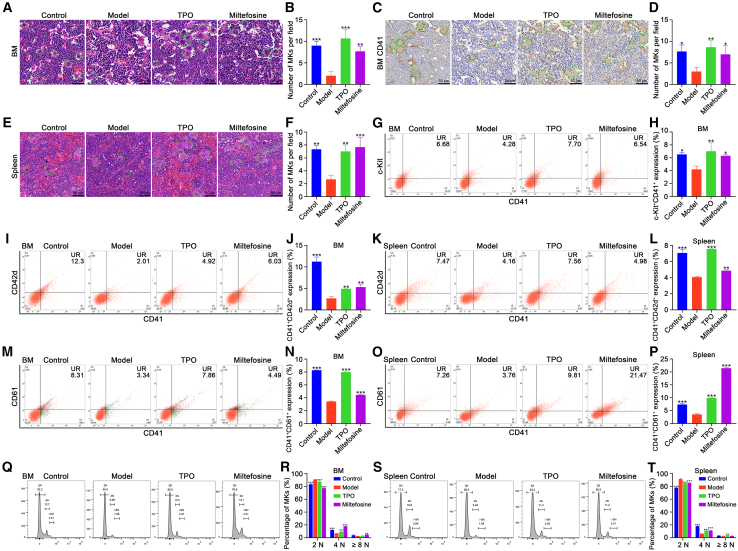
Miltefosine stimulates hematopoietic recovery in RIT mice (A) H&E staining of BM from control, model, TPO (3000 U/kg), and miltefosine (10, 20, and 40 mg/kg)-treated groups on day 10. Scale bar: 50 μM. (B) Quantification of MK counts in BM across groups. *n* = 3. (C) Immunohistochemical analysis for CD41 in BM. Scale bar, 50 μM. (D) Quantification of CD41-positive MKs in BM. *n* = 3. (E) H&E staining of spleen in each group. Scale bar, 50 μM. (F) Quantification of MK counts in spleen across groups. *n* = 3. (G and H) Flow cytometric analysis and quantification of c-Kit^+^CD41^+^ cells in BM. *n* = 3. (I and J) Flow cytometric analysis and quantification of CD41^+^CD42d^+^ cells in BM. *n* = 3. (K and L) Flow cytometric analysis and quantification of CD41^+^CD42d^+^ cells in spleen. *n* = 3. (M and N) Flow cytometric analysis and quantification of CD41^+^CD61^+^ cells in BM. *n* = 3. (O and P) Flow cytometric analysis and quantification of CD41^+^CD61^+^ cells in spleen. *n* = 3. (Q and R) Flow cytometric analysis and quantification of DNA ploidy in BM. *n* = 3. (S and T) Flow cytometric analysis and quantification of DNA ploidy in spleen. *n* = 3. Statistical significance in (B,D,F,H,J,L,N,P,R,T) was calculated using a one-way ANOVA test. ∗*p* < 0.05, ∗∗*p* < 0.01, ∗∗∗*p* < 0.001, vs. model group. Error bars represent mean ± SD.

To elucidate the reason behind the increased platelet counts observed in miltefosine-treated mice, we investigated the effects of miltefosine on megakaryopoiesis in RIT mice. Hematoxylin and eosin (H&E) staining showed an increase in MK counts in both TPO and miltefosine-treated groups relative to model group (Figures 4A and 4B). This observation was corroborated by immunohistochemical analysis for CD41, which showed elevated numbers of CD41-positive MKs in treated groups (Figures 4C and 4D), indicating that miltefosine facilitates recovery of megakaryopoiesis in the BM post-irradiation. Beyond the BM, the spleen serves as an additional site for hematopoiesis.^17^ H&E staining demonstrated that miltefosine treatment also led to an increase in MK counts within the spleen (Figures 4E and 4F). This augmentation in MK numbers is likely due to an expanded population of megakaryocytic progenitors. Flow cytometric analysis confirmed a higher percentage of c-Kit^+^CD41^+^ cells in BM, representing megakaryocytic progenitors, in both TPO and miltefosine-treated groups relative to model group (Figures 4G and 4H), suggesting that miltefosine enhances the production of these progenitors. Further assessment of MK differentiation revealed that the populations of CD41^+^CD42d^+^ and CD41^+^CD61^+^ cells were markedly increased both in BM and spleen in TPO and miltefosine-treated groups relative to model group (Figures 4I–4P). This finding aligns with our *in vitro* results, demonstrating that miltefosine effectively rescues MK differentiation in RIT mice. Furthermore, analysis of DNA ploidy revealed a shift in the MK population toward higher ploidy levels, with a concomitant decrease in 2 N cells and an increase in 4 N and ≥8 N cells in BM of miltefosine-treated mice relative to control group (Figures 4Q and 4R). Similar trends were observed in the spleen, with a decrease in 2 N cells and an increase in 4 N cells (Figures 4S and 4T). Collectively, these findings demonstrate that miltefosine effectively rescues MK production and differentiation in RIT mice.

### Gene expression profile induced by miltefosine

**Figure 5:**
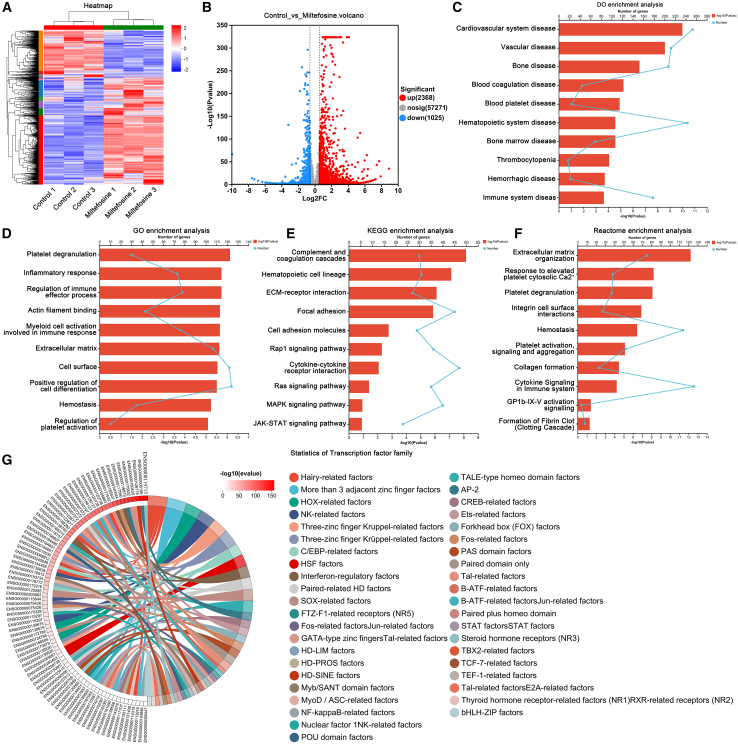
RNA sequencing reveals gene expression changes induced by miltefosine (A) Hierarchical clustering analysis of DEGs in response to miltefosine treatment. (B) Volcano plots of DEGs. (C) DO enrichment analysis of DEGs. (D) GO enrichment analysis of DEGs. (E) KEGG enrichment analysis of DEGs. (F) Reactome enrichment analysis of DEGs. (G) Identification of transcription factors from DEGs.

To investigate the molecular mechanisms by which miltefosine promotes MK differentiation, RNA sequencing was conducted on K562 cells exposed to miltefosine for three days. Hierarchical clustering analysis indicated a distinct gene expression profile between control and miltefosine-treated groups (Figure 5A). The volcano plot highlighted 2368 upregulated and 1025 downregulated genes in miltefosine-treated group relative to control (Figure 5B). Disease Ontology (DO) enrichment analysis demonstrated that differentially expressed genes (DEGs) regulated by miltefosine were predominantly associated with blood coagulation disease, blood platelet disease, hematopoietic system disease, thrombocytopenia, and immune system disease (Figure 5C). These findings are closely aligned with the thrombocytopenia context explored in this study. Gene Ontology (GO) enrichment analysis demonstrated that DEGs were involved in processes including myeloid cell activation involved in immune response, positive regulation of cell differentiation, hemostasis, and regulation of platelet activation (Figure 5D). These functions align with the observed phenotype of miltefosine on promoting MK differentiation and enhancing platelet coagulation. Kyoto Encyclopedia of Genes and Genomes (KEGG) pathway analysis further suggested enrichment of DEGs in pathways including complement and coagulation cascades, hematopoietic cell lineage, Ras, MAPK, and JAK-STAT signaling pathways (Figure 5E). These pathways are known to regulate MK differentiation and thrombopoiesis.^4^^,^^6^^,^^18^ Reactome pathway analysis corroborated these findings, showing that DEGs were enriched in pathways related to platelet degranulation, hemostasis, platelet activation, signaling and aggregation, and cytokine signaling in immune system (Figure 5F). These enrichments are consistent with the observed effects of miltefosine in RIT mice. Transcription factors were identified within the DEGs using the JASPAR database. Notably, transcription factors from GATA-type zinc finger, TAL-related factors, STAT factors, and Kruppel-related three-zinc finger families were found (Figure 5G). Many of these transcription factors such as TAL1 and EGR1 have been previously implicated in MK differentiation and thrombopoiesis,^19^^,^^20^^,^^21^ underscoring their potential roles in the molecular mechanisms activated by miltefosine.

### Miltefosine directly binds to CCR5

**Figure 6:**
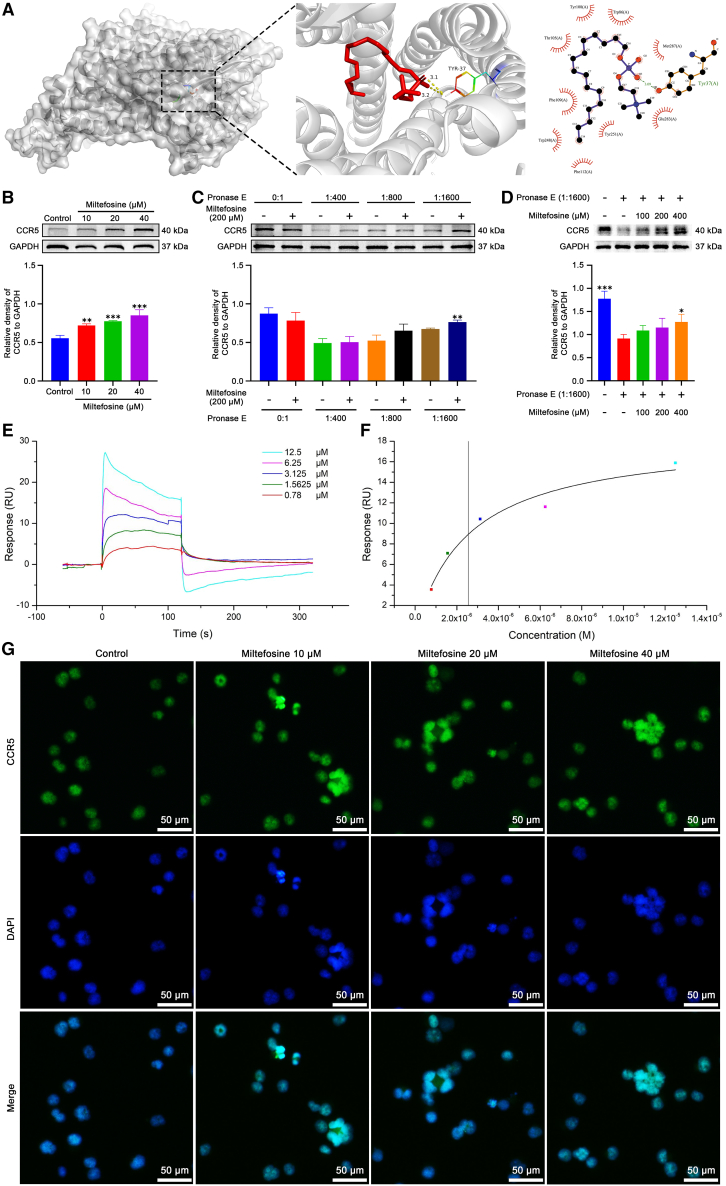
Miltefosine directly interacts with CCR5 (A) Molecular docking analysis of miltefosine interaction with CCR5. (B) Western blot analysis of CCR5 expression after treatment with miltefosine (10, 20, and 40 μM) for 5 days *n* = 3. (C) DARTS assay demonstrating miltefosine binding to CCR5 under varying concentrations of pronase E (1:400, 1:800, 1:1600) and miltefosine (200 μM). *n* = 3. (D) DARTS assay with different concentrations of miltefosine (100, 200, 400 μM) and pronase E (1:1600). *n* = 3. (E) Sensorgram showing the binding kinetics of miltefosine to CCR5 at different concentrations (0.78, 1.5625, 3.125, 6.25, and 12.5 μM) measured by SPR. (F) Binding curve representing the saturation model fitted to the experimental SPR data. (G) Immunofluorescence staining of CCR5 in cells treated with miltefosine (10, 20, and 40 μM) for 5 days. Scale bar: 50 μM. Data represent mean ± SD of three independent experiments. ∗*p* < 0.05, ∗∗*p* < 0.01, ∗∗∗*p* < 0.001, vs. corresponding control group. Statistical significance in (B–D) was calculated using a one-way ANOVA test. ∗*p* < 0.05, ∗∗*p* < 0.01, ∗∗∗*p* < 0.001, vs. control group, Error bars represent mean ± SD.

To identify the direct target of miltefosine, we utilized molecular docking to screen potential targets upstream of the signaling pathways identified in our RNA sequencing analysis. Among the candidates, CCR5 demonstrated a high binding affinity with miltefosine (6.0 kcal/mol) (Figure 6A). Western blot analysis showed that miltefosine caused a dose-dependent increase in CCR5 level (Figures 6B and S5). To validate the binding interaction between miltefosine and CCR5, we employed the drug affinity responsive target stability (DARTS) assay. Following incubation of K562 cell lysates with miltefosine (200 μM), we observed higher CCR5 protein levels in miltefosine-treated groups relative to untreated controls, across varying concentrations of protease E (1:400, 1:800, or 1:1600) (Figures 6C and S5). Furthermore, under the dilution of pronase E (1:1600), miltefosine conferred increased resistance to pronase E-induced degradation of CCR5 in a concentration-dependent manner (Figures 6D and S5). The surface plasmon resonance (SPR) was further employed to validate the binding of miltefosine to CCR5. CCR5 protein was immobilized on a CM5 sensor chip, and miltefosine was injected in concentrations ranging from 0.78 μM to 12.5 μM. The sensorgram reveals a dose-dependent binding response, with higher concentrations of miltefosine inducing stronger binding (Figure 6E), indicating a specific and concentration-dependent interaction. The association phase shows a rapid increase in response units (RU), followed by a dissociation phase after the analyte washout (Figure 6E). The equilibrium binding data were fitted to a saturation model, yielding a dissociation constant (K_D_) of 2.55 μM (Figure 6F), suggesting a high affinity between miltefosine and CCR5. Immunofluorescence assays further demonstrated that miltefosine enhances CCR5 expression, particularly in mature MKs (Figure 6G). Collectively, these data indicate that miltefosine directly binds to CCR5, suggesting that CCR5 may serve as a key target mediating the effects of miltefosine on MK differentiation.

### Miltefosine regulates the expression of proteins related to MAPK and JAK2/STAT signaling pathway and hematopoietic transcription factors

**Figure 7:**
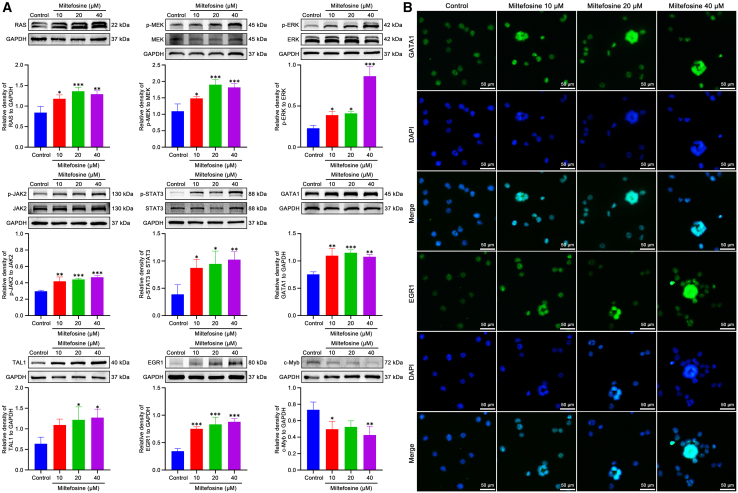
Miltefosine modulates signaling pathways and transcription factors involved in MK differentiation (A) Western blot analysis of MAPK and JAK2/STAT signaling pathway proteins and hematopoietic transcription factors in cells treated with miltefosine (10, 20, and 40 μM) for 5 days. *n* = 3. Statistical significance was calculated using a one-way ANOVA test. ∗*p* < 0.05, ∗∗*p* < 0.01, ∗∗∗*p* < 0.001, vs. control group. Error bars represent mean ± SD. (B) Immunofluorescence staining of hematopoietic transcription factors in cells treated with miltefosine (10, 20, and 40 μM) for 5 days.

To elucidate the pathways regulated by miltefosine during MK differentiation, we assessed the expression of key proteins from various pathways identified in our RNA sequencing analysis. Western blot revealed that miltefosine upregulated expression of RAS and increased phosphorylation levels of MEK and ERK (Figure 7A; Figure S6), all of which are components of the MAPK signaling pathway. Additionally, miltefosine enhanced the phosphorylation of JAK2 and STAT3 (Figures 7A and S6), indicating activation of the JAK2/STAT signaling pathway. We also examined expression of hematopoietic transcription factors identified through RNA sequencing. Miltefosine treatment upregulated the expression of GATA1, TAL1, and EGR1, while downregulating c-Myb (Figures 7A and S6). Immunofluorescence assays confirmed the upregulation of GATA1, TAL1, and EGR1, particularly in mature and high-ploidy MKs (Figure 7B). Moreover, the expression of proteins of the BM cells from the RIT mouse model was also detected. The results showed that the phosphorylation levels of MEK and ERK, as well as JAK2 and STAT3, were significantly lower in the model group compared to the control group (Figures S7 and 8). However, the TPO and miltefosine-treated groups exhibited markedly higher phosphorylation levels than the model group (Figures S7 and 8). These results suggest that miltefosine effectively counteracts the radiation-induced reduction in phosphorylation of MEK, ERK, JAK2, and STAT3. Taken together, above findings demonstrate that miltefosine activates the MAPK and JAK2/STAT pathways, along with their downstream transcription factors.

**Figure 8:**
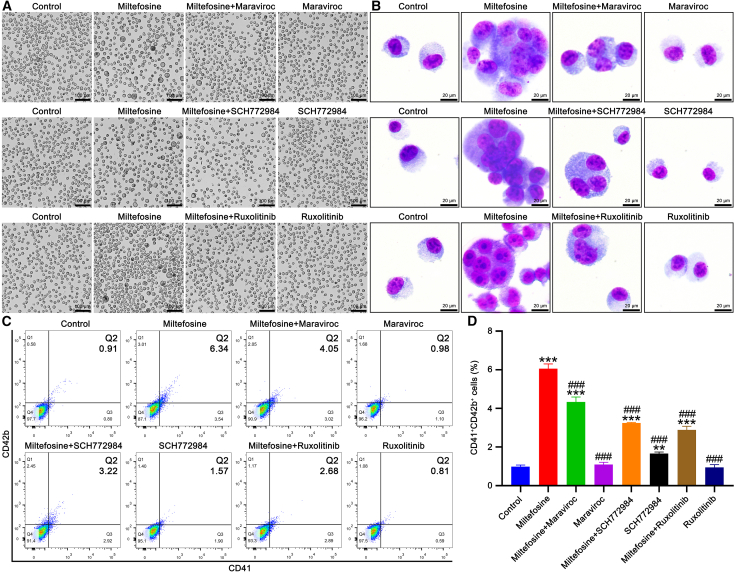
CCR5, MAPK, and JAK2/STAT3 signaling pathways mediate miltefosine-induced MK differentiation (A) Representative bright-field images of K562 cells treated with miltefosine (40 μM), maraviroc (1 μM), SCH772984 (1 μM), or ruxolitinib (0.5 μM) for 5 days. Scale bar: 100 μM. (B) Giemsa staining of K562 cells after indicated treatments. Scale bar: 20 μM. (C and D) Flow cytometric analysis and quantification of CD41^+^CD42b^+^ cells after indicated treatments. *n* = 3. Statistical significance was calculated using a one-way ANOVA test. ∗∗*p* < 0.01, ∗∗∗*p* < 0.001, vs. control group. ^###^*p* < 0.001, vs. miltefosine-treated group. Error bars represent mean ± SD.

### Miltefosine promotes MK differentiation by activation of CCR5/MAPK and CCR5/JAK2/STAT3 signaling pathways

**Figure 9:**
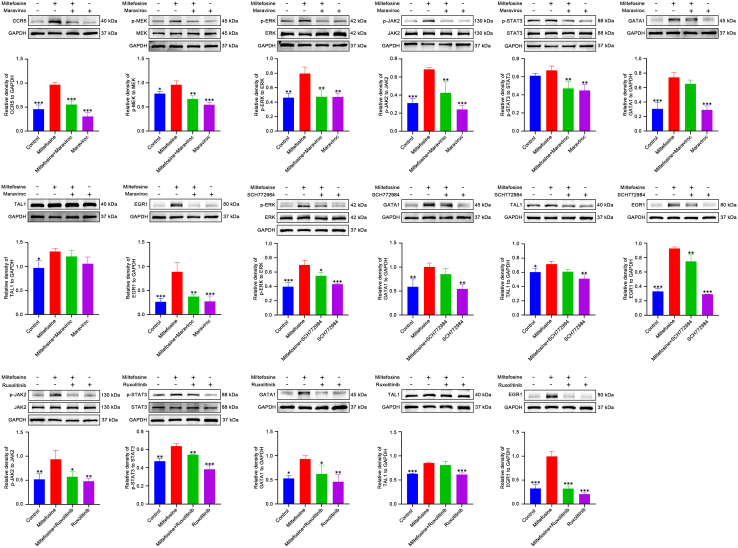
Western blot analysis reveals the regulatory relationships among miltefosine, CCR5, MAPK, JAK2/STAT3 signaling pathways, and transcription factors Statistical significance was calculated using a one-way ANOVA test. *n* = 3. ∗*p* < 0.05, ∗∗*p* < 0.01, ∗∗∗*p* < 0.001, vs. miltefosine-treated group. Error bars represent mean ± SD.

**Figure 10:**
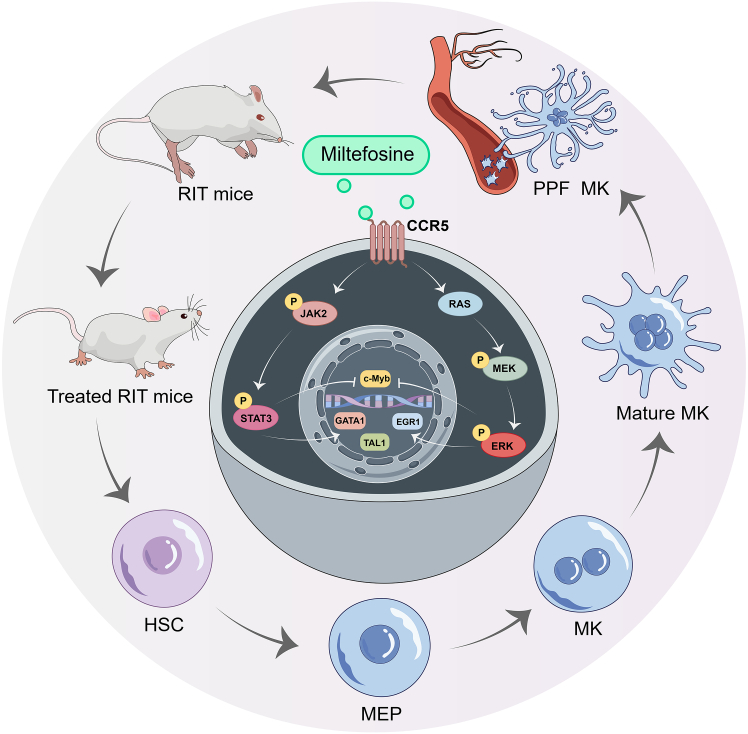
Molecular mechanisms underlying miltefosine regulation of MK differentiation and thrombopoiesis

To confirm the role of CCR5, as well as the MAPK and JAK2/STAT3 pathways, in mediating miltefosine-induced MK differentiation, we employed specific inhibitors: maraviroc (CCR5 inhibitor), SCH772984 (ERK inhibitor), and ruxolitinib (JAK2 inhibitor). Our findings revealed that maraviroc, SCH772984, and ruxolitinib partially inhibited the miltefosine-induced formation of large, multinucleated cells (Figures 8A and 8B) and the increase in the proportion of CD41^+^CD42b^+^ cells (Figures 8C and 8D). To investigate hierarchical connection between CCR5, MAPK, JAK2/STAT3 pathways, GATA1, TAL1, and EGR1, we assessed the expression of relevant proteins following treatment with the inhibitors. Maraviroc treatment significantly attenuated the miltefosine-induced upregulation of CCR5, phosphorylation of MEK, ERK, JAK2, and STAT3, and expression of GATA1 and EGR1 (Figures 9 and S9). TAL1 expression also exhibited a decreasing trend with maraviroc treatment (Figures 9 and S9). SCH772984 treatment significantly reduced the miltefosine-induced upregulation of ERK phosphorylation and EGR1 expression (Figure 9; Figure S9). Additionally, co-treatment with miltefosine and SCH772984 resulted in a trend toward decreased GATA1 and TAL1 expression compared to miltefosine alone (Figure 9; Figure S9). Similarly, Ruxolitinib treatment markedly suppressed the miltefosine-induced upregulation of JAK2 and STAT3 phosphorylation, as well as GATA1 and EGR1 expression (Figure 9; Figure S9). TAL1 expression also showed a decreasing trend with miltefosine and ruxolitinib co-treatment compared to miltefosine alone (Figure 9; Figure S9). Collectively, these results demonstrate that miltefosine promotes MK differentiation through activating CCR5/MAPK and CCR5/JAK2/STAT3 signaling pathways (Figure 10).

## Discussion

Cancer remains a leading global health challenge with rising incidence and mortality rates. Despite the advent of innovative therapies such as targeted and immunotherapies, radiation therapy continues to be a cornerstone in cancer treatment.^9^^,^^10^ While radiation therapy effectively combats cancer, its deleterious impact on hematopoietic tissue, characterized by rapid cellular turnover, is well-established. Ionizing radiation directly and indirectly inflicts DNA damage, compromising hematopoietic progenitor and immature cells within the BM. Subsequent depletion of mature blood cells results in BM suppression and RIT. This thrombocytopenic state heightens the risk of bleeding and infection, significantly compromising patient survival. Moreover, RIT necessitates dose reductions or delays in radiation therapy, further jeopardizing patient outcomes.^9^^,^^10^^,^^22^ Currently, therapeutic options for RIT are limited. Platelet transfusions, although rapidly restoring platelet counts, are associated with substantial risks including severe immune reactions, thrombosis, and acute lung injury.^23^ TPO-RAs offer an alternative to rhTPO by circumventing immune-related complications. However, TPO-RAs are associated with excessive platelet elevation, predisposing patients to thrombosis and potentially increasing the incidence of malignancies such as leukemia and myeloma.^11^^,^^12^ Moreover, some patients develop resistance to TPO-RAs, and these agents are ineffective in patients with c-Mpl gene deficiencies.^24^^,^^25^ Given these limitations, the development of safe and effective, non-TPO-RA-based therapies for RIT is imperative.

Miltefosine, a phosphatidylcholine synthesis inhibitor initially designed as a chemotherapeutic agent, has demonstrated potent antiparasitic activity and is currently the sole oral medication approved for the treatment of Leishmaniasis and Chagas disease.^13^ Beyond its established role in antiparasitic therapy, miltefosine has shown promise as an antifungal and antibacterial agent.^26^^,^^27^ Preclinical data in rodents suggest that miltefosine does not induce immunosuppression or hematotoxicity at therapeutic doses.^15^^,^^16^ Furthermore, in a clinical study involving 72 patients with various cancers, oral administration of miltefosine for a median duration of six weeks resulted in a notable hematological response. Specifically, approximately 73% of patients experienced a 1.19- to 2.33-fold platelet count elevation.^14^ These findings suggest a thrombocytopenic ability of miltefosine in cancer patients, prompting exploration of its therapeutic utility in RIT.

We have demonstrated that promoting MK differentiation is conducive to platelet production.^9^^,^^28^ To explore effects of miltefosine on MK differentiation, we employed K562 and HEL cells, two well-established cell lines for investigating MK differentiation.^9^^,^^28^ Our results indicated that miltefosine significantly promoted MK differentiation. This was evidenced by increased cell size, multinucleation, β-tubulin reorganization, DNA ploidy, and upregulated CD41, CD42b, and CD61 expressions.

Following confirmation of miltefosine’s activity *in vitro*, we evaluated its therapeutic effects on RIT. Miltefosine administration significantly accelerated the recovery of platelet counts in irradiated mice, achieving levels that were restored to normal without surpassing physiological limits. In contrast, treatment with TPO, a positive control, resulted in abnormally high platelet counts, posing a risk of adverse reactions. Therefore, miltefosine may avoid excessive thrombocytosis and the associated risk of thrombosis observed with rhTPO and TPO-RAs. Moreover, miltefosine did not affect MPV, organ indices, or structure, nor did it significantly impact serum biochemical markers such as ALT, AST, and CREA in irradiated mice. Interestingly, low-dose miltefosine (10 mg/kg) notably reduced the elevated BUN levels caused by irradiation, suggesting a protective effect against irradiation toxicity. Elevating platelet numbers alone is insufficient; functional hemostasis is critical to prevent life-threatening bleeding. To assess the functionality of miltefosine-induced platelets, we evaluated their hemostatic capacity. In a carotid artery thrombosis model, miltefosine-treated irradiated mice displayed enhanced thrombus formation at the site of FeCl_3_-induced injury, indicating efficient hemostasis. Platelet aggregation is a critical step in hemostasis, wherein circulating anucleate cells adhere and coalesce at sites of vascular injury. Upon activation by agonists including ADP, serotonin, epinephrine, thrombin, and collagen, platelets undergo shape change and granule release, culminating in forming hemostatic plug. Clinical significance of assessing platelet aggregation lies in its relevance to evaluating hemostatic function and thrombotic risk. Our experiments demonstrated that miltefosine enhanced platelet aggregation after ADP and collagen stimulation. Furthermore, in a tail bleeding assay, miltefosine effectively promoted timely hemostasis following tail amputation in irradiated mice. These findings collectively support the notion that miltefosine-induced platelets possess robust hemostatic function. These findings suggest that miltefosine not only effectively enhances platelet levels and function without *in vivo* toxicity but also aligns with clinical observations where it significantly increased platelet counts in cancer patients without causing myelotoxicity.

Enhanced MK production and differentiation have been implicated as potential mechanisms underlying increased platelet counts. Given the pivotal role of the BM in hematopoiesis, we evaluated MK generation through morphological analysis. We found that miltefosine significantly increased MK counts in BM of RIT mice, suggesting enhanced megakaryopoiesis. Further analysis of megakaryocytic progenitors demonstrated that miltefosine markedly promoted the proportion of megakaryocytic progenitors, providing a direct explanation for the increased number of MKs. Additionally, we examined MK differentiation and found that miltefosine significantly promoted both MK differentiation and ploidy in the BM, indicating that miltefosine not only stimulated megakaryopoiesis but also promoted the further differentiation of these MKs into platelets. Beyond the BM, the spleen also plays a significant role in hematopoiesis.^17^ Our results showed that miltefosine exerted similar effects on MKs in the spleen, with increases observed in both number and differentiation. These findings indicate that the elevation of platelet levels in irradiated mice treated with miltefosine is attributed to the enhancement of hematopoietic function in both the BM and spleen.

RNA sequencing has revealed the molecular mechanisms by which miltefosine regulates MK differentiation. DO enrichment analysis, a bioinformatics method used to identify enrichment of target genes in specific diseases or disease categories, was employed to uncover potential gene-disease associations and understand the functional roles of genes in disease contexts. Our DO analysis showed that DEGs regulated by miltefosine were significantly enriched in platelet-related diseases, particularly thrombocytopenia, blood coagulation disease, blood platelet disease. This suggests that miltefosine influences the expression of pathological genes associated with platelet quantity or functional abnormalities, consistent with its demonstrated efficacy. Interestingly, cardiovascular system disease and vascular disease were the most significantly enriched in the DO analysis. Previous studies have demonstrated that miltefosine can ameliorate atherosclerosis by promoting cholesterol efflux from macrophages, inhibiting NLRP3 inflammasome activation, and enhancing autophagy and mitophagy.^29^ Furthermore, miltefosine improves reverse cholesterol transport, reduces inflammation, and modulates the gut microbiota.^30^ Collectively, these mechanisms contribute to the reduction of lipid accumulation and plaque formation, thereby protecting against the progression of atherosclerosis.^29^^,^^30^ Of particular note is the relationship between thrombocytopenia and cardiovascular diseases. Thrombocytopenia is a recognized risk factor for cardiovascular conditions and is associated with higher mortality.^31^ Given that miltefosine demonstrates therapeutic potential for both thrombocytopenia and cardiovascular diseases such as atherosclerosis, our findings suggest that its ability to promote platelet recovery could have significant implications for improving patient outcomes in cardiovascular disease. By addressing both platelet-related abnormalities and the pathophysiology of cardiovascular conditions, miltefosine offers a dual therapeutic approach with the potential to benefit patients suffering from both thrombocytopenia and cardiovascular diseases. This highlights the broader therapeutic value of miltefosine, not only as an agent for platelet recovery but also as a potential treatment strategy for managing cardiovascular diseases, particularly those complicated by thrombocytopenia. Future studies exploring this dual efficacy could provide new insights into the development of more comprehensive treatments for patients with these coexisting conditions. Subsequent GO, KEGG, and Reactome enrichment analyses further indicated that miltefosine modulated genes involved in MK differentiation, platelet production, and hemostatic function. Notably, the MAPK and JAK2/STAT signaling pathway were closely associated with these processes. Given the regulation of these pathways by multiple factors and their receptors, we used molecular docking to predict potential protein receptors that miltefosine might regulate. The results revealed a high binding affinity between miltefosine and CCR5. CCR5 is expressed on immune cells. It plays a pivotal role in recruiting these cells to sites of inflammation.^32^ CCR5 is also known as a major co-receptor for HIV-1 entry into host cells, with mutations like CCR5Δ32 conferring resistance to HIV infection.^32^^,^^33^ Moreover, CCR5 is aberrantly expressed in various cancers, including breast and prostate cancers, contributing to tumorigenesis and progression.^34^ Binding of CCL5 to CCR5 activates downstream signalings, such as MAPK, JAK2/STAT, PI3K/Akt, and TGF-β/Smad, influencing metastasis, inflammation, angiogenesis, invasion, and division.^7^^,^^32^ While researchers are primarily focused on finding effective CCR5 inhibitors to treat diseases caused by abnormal expression of CCL5 or CCR5, such as maraviroc-the first FDA-approved drug for HIV treatment, also used in some anticancer therapies-the CCL5/CCR5 axis plays a positive regulatory role in hematopoiesis.^7^^,^^32^^,^^35^ Studies have shown that platelets store inflammatory cytokines, including CCL5, which are released upon stimulation by factors like ADP and thromboxane A2. CCL5 binds to CCR5 on MKs, activating the downstream Akt pathway, promoting MK maturation, and increasing platelet production. Maraviroc, however, blocks this effect.^7^ Another study found that BM endothelial cells release CCL5, and following irradiation in mice, the expression of CCL5 and CCR5 in the BM increases. The CCL5 released by endothelial cells binds to CCR5 on hematopoietic cells, enhancing their cycling and survival, thereby promoting hematopoietic regeneration and counteracting irradiation-induced myelosuppression. This indicates that CCR5 is a therapeutic target for hematopoietic regeneration following irradiation injury.^35^ To directly assess the interaction between miltefosine and CCR5, we performed DARTS and SPR assays, which demonstrated a specific binding between these molecules. Importantly, pharmacological inhibition of CCR5 with maraviroc abolished miltefosine-induced MK differentiation, unequivocally establishing CCR5 as the direct target of miltefosine in promoting MK differentiation. Our findings highlighting the role of CCR5 in MK differentiation, platelet production, and hematopoietic recovery, position CCR5 as a promising therapeutic target for RIT. However, due to the absence of CCR5 knockout mice in our study, we were unable to perform further *in vivo* validation of miltefosine’s therapeutic efficacy specifically in CCR5-deficient models. This limitation underscores the need for additional research using CCR5 knockout mice to more comprehensively explore the precise role of CCR5 in mediating the effects of miltefosine. Future studies addressing this gap could provide valuable insights into the underlying mechanisms and further substantiate the therapeutic potential of miltefosine in treating thrombocytopenia, particularly in CCR5-targeted therapies.

To further elucidate the downstream signaling pathways activated by CCR5 upon miltefosine binding, we examined the expression of key signaling molecules. While previous studies have implicated the Akt pathway in regulating MK differentiation and thrombopoiesis, we found that miltefosine did not significantly affect PI3K and Akt expression (data not shown). This finding is intriguing given the established role of Akt as a proto-oncogene and a therapeutic target in various cancers.^36^ Miltefosine itself is an approved Akt inhibitor but is used for the treatment of leishmaniasis, not cancer.^13^^,^^36^ Conversely, Akt plays a positive role in MK differentiation.^7^^,^^37^^,^^38^ The apparent discrepancy between the inhibitory influence of miltefosine on Akt in cancer cells and its lack of effect on Akt in MKs, coupled with its stimulatory effect on MK differentiation, suggests a complex and context-dependent mechanism of action. Indeed, inhibition of Akt in MKs would be expected to impair MK differentiation and thrombopoiesis. Given the unexpected finding regarding Akt, we next focused on the MAPK and JAK/STAT signaling pathways, which were also identified as potential downstream effectors of CCR5 in our RNA-seq analysis. We observed that miltefosine activated key signaling molecules within both the MAPK and JAK2/STAT pathways. Furthermore, pharmacological inhibition of either the MAPK or JAK2/STAT pathway significantly attenuated miltefosine-induced MK differentiation. Importantly, blockade of CCR5 with maraviroc also suppressed activation of MAPK and JAK2/STAT pathways in response to miltefosine. Taken together, our data provide compelling evidence that miltefosine promotes MK differentiation through a CCR5-dependent mechanism involving the activation of MAPK and JAK2/STAT signaling.

To further elucidate the transcriptional mechanisms downstream of the MAPK and JAK2/STAT signaling pathways, we employed the JASPAR database to identify transcription factors within our DEG dataset. Among the identified transcription factors, EGR1, TAL1, and GATA1 emerged as key regulators. EGR1, an early growth response gene, has been well-characterized for its rapid induction in response to vascular injury. Notably, EGR1 is a direct downstream target of the ERK, and the MEK/ERK/EGR1 axis has been implicated in cardiovascular disease.^39^ Previous studies have shown that the polymethoxyflavone nobiletin stimulates MK differentiation in an MAPK/ERK-dependent manner through upregulation of EGR1.^20^ Consistent with these findings, we found an increase in EGR1 expression in response to miltefosine treatment. Importantly, this effect was abrogated by pharmacological inhibition of CCR5, MAPK, or JAK2/STAT signaling, indicating that EGR1 functions downstream of the CCR5/MAPK and CCR5/JAK2/STAT axis. Moreover, we found that TAL1, another known megakaryocytic transcription factor,^19^ exhibited a similar expression pattern as EGR1 in response to miltefosine. In contrast, c-Myb, a negative regulator of MK differentiation,^40^ was significantly downregulated by miltefosine. Furthermore, the expression of GATA1, a master regulator of MK differentiation,^40^^,^^41^ was also upregulated in response to miltefosine and correlated with the expression of EGR1 and TAL1. Collectively, our data suggest that EGR1, TAL1, GATA1, and c-Myb are key transcription factors downstream of the miltefosine-CCR5-MAPK-JAK2/STAT signaling axis, orchestrating the process of MK differentiation (Figure 10).

Our findings establish CCR5 as a critical enhancer of MK differentiation and thrombopoiesis. By demonstrating that miltefosine, a CCR5 agonist, promotes MK differentiation and platelet formation through activation of MAPK and JAK2/STAT signaling, we unveil a therapeutic axis for thrombocytopenia. Our study highlights the potential of CCR5-targeted therapies as an alternative or complementary approach to TPO-RAs for treating thrombocytopenia. This study thus provides a mechanistic framework for developing innovative strategies to address this clinically challenging hematological disorder.

### Limitations of the study

This study identifies a therapeutic strategy for thrombocytopenia and provides a foundation for exploring CCR5-targeted interventions in a wider range of thrombocytopenic conditions. However, the therapeutic effect of miltefosine on CCR5 knockout mice was not investigated in this study. Such an investigation would not only further confirm CCR5 as a viable therapeutic target for thrombocytopenia but also provide *in vivo* evidence that miltefosine promotes thrombocytopoiesis by targeting CCR5.

## Resource availability

### Lead contact

Further information and requests for resources and reagents should be directed to and will be fulfilled by the lead contact, Long Wang (wanglong1226@swmu.edu.cn).

### Materials availability

This study did not generate new unique reagents.

### Data and code availability

• RNA-seq data have been deposited at SRA and are publicly available as of the date of publication. Accession numbers are listed in the key resources table. Original western blot images have been deposited at Mendeley and are publicly available as of the date of publication. The DOI is listed in the key resources table. Microscopy data reported in this paper will be shared by the lead contact upon request.
• The article does not report any original code.
• Any additional information required to reanalyze the data reported in this paper is available from the lead contact upon request.

## Acknowledgments

This work was supported by the 10.13039/501100001809National Science Foundation of China (82204666, 82074129, 81774013, and 82273889), the 10.13039/501100018542Sichuan Provincial Natural Science Foundation General Project (2024NSFSC0711 and 2023NSFSC0657), the Joint Project of Luzhou Municipal People’s Government and Southwest Medical University (2024LZXNYDJ030), the Science and Technology Program Joint Innovation Project of Sichuan Province (2022YFS0635 and 2022YFS0635-B1), the Sichuan Outstanding Youth Fund Project (2022JDJQ0061), and the National Innovation and Entrepreneurship Training Program for College Students of China (202410632006, 202210632031, 202310632102, and S202310632303).

## Author contributions

Conceptualization, L.W. and J.W.; methodology, L.W., Q.L., T.Z., Z.L., and X.Q; software, X.M., S. Liu, and S.H.; validation, L.W., Q.L., T.Z., Z.L., and X.Q.; formal analysis, G.Q., R.L., and H.S.; investigation, L.W., Q.L., T.Z., Z.L., and X.Q; resources, L.W., J.L., and J.W.; data curation, L.W. and J.L.; writing—original draft preparation, L.W.; writing—review and editing, L.W. and J.W.; visualization, J.Z., F.H., S.D., S. Li, and J.L.; supervision, L.W. and J.W.; project administration, L.W. and J.W.; funding acquisition, L.W. and J.W. All authors have read and agreed to the published version of the manuscript.

## Declaration of interests

The authors declare no competing interests.

## STAR★Methods

### Key resources table

**Table:** 

REAGENT or RESOURCE	SOURCE	IDENTIFIER
**Antibodies**		

CCR5 Antibody	Abmart	Cat# TA6339
Ras (E8N8L) XP® Rabbit	CellSignaling	Cat# 67648S
Phospho-MEK1/2 (Ser217/221) (41G9) Rabbit mAb	CellSignaling	Cat# 9154T
MEK1/2 (L38C12) Mouse mAb	CellSignaling	Cat# 4694S
Phospho-ERK1/2 (Thr202/Tyr204) Polyclonal antibody	Proteintech	Cat# 28733-1-AP
ERK1/2 Monoclonal antibody	Proteintech	Cat# 66192-1-Ig
Phospho-STAT3 (Ser727) Polyclonal antibody	Proteintech	Cat# 28945-1-AP
STAT3 Polyclonal antibody	Proteintech	Cat# 10253-2-AP
MYB/c-Myb Polyclonal antibody	Proteintech	Cat# 17800-1-AP
TAL1 Antibody	CellSignaling	Cat# 12831S
Phospho-JAK2 (Y1007 + Y1008) Antibody	Abmart	Cat# 56570
JAK2 Antibody	Abmart	Cat# TA6022
Egr1 Antibody	Abmart	Cat# T57177M
Anti-GAPDH antibody	Abcom	Cat# ab8245
Anti-GATA1 antibody	Abcom	Cat# ab308028
FITC Anti-Human CD41 Antibody	Elabscience	Cat# E-AB-F1088C
PE Anti-Human CD61 Antibody	Elabscience	Cat# E-AB-F1166D
CD117 (c-Kit) Monoclonal Antibody	Thermo Fisher Scientific	Cat# 12-1171-83
CD42d Monoclonal Antibody	Thermo Fisher Scientific	Cat# 14-0421-82
CD61 (Integrin beta 3) Monoclonal Antibody	Thermo Fisher Scientific	Cat# 12-0619-42
FITC Anti-Mouse CD41 Antibody	Elabscience	Cat# E-AB-F1183C
PE Anti-Mouse CD62P Antibody	BioLegend	Cat# 148306

**Chemicals, peptides, and recombinant proteins**		

Miltefosine	APExBIO	Cat# B1371
RIPA lysis buffer	Beyotime	Cat# P0013B
Triton X-100	zsbio	Cat# ZLI-9308
DMSO	solarbio	Cat# D8371
KCl	Sinopharm	Cat# 10016318
Methanol	Fisher Scientific	Cat# A456-4
DAPI solution	Solarbio	Cat#C0065

**Critical commercial assays**		

Cell Counting Kit-8	Dojindo, Kyushu, Japan	Cat# CK04
Giemsa Staining Solution	Beyotime	Cat# C0133
TRITC Phalloidin	Solarbio	Cat#CA1610
Mitochondrial membrane potential assay kit with JC-1	Beyotime	Cat# C2006
Mito-Tracker Green	Beyotime	Cat# C1048
Biotinylated Human CCR5 Protein	KACTUS	Cat# CR5-HM4N191B

**Deposited data**		

RNA-seq data	This paper	SRA: PRJNA1152712
Original western blot	This paper	Mendeley:https://data.mendeley.com/datasets/8rrt786c3d/1

**Experimental models: Cell lines**		

K562	American Type Culture Collection	Cat#CRL-3344
HEL	American Type Culture Collection	Cat#TIB-180

**Experimental models: Organisms/strains**		

Kunming mice	Da Shuo Biotechnology	N/A

**Software and algorithms**		

Prism 8	GraphPad Software	https://www.graphpad.com/
FlowJo, version 10.7.2	BD Biosciences	https://www.flowjo.com/solutions/flowjo/downloads
ImageJ	FIJI	https://imagej.net/

### Experimental model and study participant details

#### Cell culture

K562 and HEL cell lines were purchased from American Type Culture Collection (ATCC, Bethesda, MD, USA), which were maintained in RPMI-1640 medium (Procell, Wuhan, China) with 10 % fetal bovine serum and 1 % penicillin/streptomycin (Merck Millipore, Darmstadt, Germany). Cultured in a 37°C incubator containing 5% CO_2._

#### Animals

Four-week-old, Half male and half female Kunming (KM) mice were purchased from Da Shuo Biotechnology Co., Ltd (Chengdu, China). The animals were randomly grouped by sex and bred under standard laboratory conditions. *In vivo* experiments were carried out following protocols authorized by laboratory animal ethics committee of Southwest Medical University (Luzhou, China, License No. 20220228-024).

### Method details

#### Chemicals

Miltefosine (purity = 98%, HPLC) was obtained from APExBIO (Houston, TX, USA).

#### CCK-8 assay

A total of 5.0 × 10^3^ cells were seeded and incubated with miltefosine (2, 5, 10, 20, 40, 80, 160, 320 μM) for 1, 3, and 5 days. Subsequently, the samples were incubated with Cell Counting Kit-8 (CCK-8) solution (Dojindo, Kyushu, Japan) for 1 h at 37°C. The 450 nm absorbance was detected by a microplate reader (BioTek, IL, USA).

#### Morphological analysis

Cells were treated with miltefosine (10, 20, and 40 μM) or phorbol 12-myristate 13-acetate (PMA, 0.8 nM; Selleck, Houston, TX, USA) for 5 days. Cellular was subsequently examined by microscopy (Leica, Wetzlar, Germany).

#### Giemsa staining

Cells were treated using a 3:1 methanol and acetic acid (v/v) for 5 min, then cytospun onto slides and incubated with 1 × Giemsa Staining Solution (Beyotime, Shanghai, China). Observations and imaging were performed by microscopy (Leica, Wetzlar, Ger many).

#### Phalloidin staining

Samples were fixed in 4% formaldehyde for 15 min on ice and permeabilized with 0.5% Triton X-100 for 10 min at 37°C. After cytospinning onto slides, samples were treated with Rhodamine-Phalloidin (UElandy, Suzhou, China). Cellular morphology was examined by fluorescence microscopy (Leica, Germany).

#### Measurement of MK differentiation and ploidy *in vitro*

Cells were exposed to miltefosine at concentrations of 10, 20, and 40 μM, PMA (0.8 nM), maraviroc (1 μM), SCH772984 (1 μM), or ruxolitinib (0.5 μM) for 5 days. For MK differentiation analysis, cells were stained by FITC-CD41 (Elabscience, Wuhan, China), PE-CD42b (Thermo Fisher Scientific, Waltham, MA, USA), or PE-CD61 (Elabscience, Wuhan, China) antibodies. Proportion of CD41^+^CD42b^+^ cells and CD41^+^CD61^+^ cells were determined by flow cytometry (Beckman Coulter, California, Brea, USA). To assess MK ploidy, cells were treated with 70% ethanol for 30 min at 4°C, incubated with PI (Beyotime, Shanghai, China) for 30 min in the dark, and detected by flow cytometry (Beckman Coulter, California, Brea, USA).

#### MMP detection

MMP was assessed using the Mitochondrial membrane potential assay kit with JC-1 (Beyotime, Shanghai, China) according to the manufacturer’s instructions.

#### Mitochondrial mass measurement

Mitochondrial mass was measured using Mito-Tracker Green (Beyotime, Shanghai, China) following the provided protocol.

#### RIT mouse model

Mice were acclimatized for one week before being divided into control, model, TPO (3000 U/kg), and miltefosine (10, 20, and 40 mg/kg) groups. All groups except control received irradiation of 4 Gy X-ray to establish the RIT model. Starting one day post-irradiation, control and model groups were administered with normal saline through intraperitoneal injections daily, while treatment groups received intraperitoneal injections of TPO (3000 U/kg) or miltefosine at indicated doses for 16 consecutive days.

#### Hematological analysis

Blood was collected from fundus vein plexus of each mouse, diluted with diluent, and subjected to hematological analysis.

#### Visceral index

After 16 days of treatment, major organs were harvested, rinsed in saline, blotted dry, and weighed. The visceral index was deter.

#### Measurement of serum biochemical indicator

Blood was collected via eye enucleation and serum was isolated. Serum levels of ALT, AST, CREA, and BUN were determined by automated biochemistry analyzer (Hitachi 7600).

#### Histology analysis and immunohistochemical staining

Femurs and spleens were collected on day 10, and heart, liver, and lung tissues were harvested on day 16. All tissues were preserved in 10% paraformaldehyde, while femurs were decalcified for over 30 days prior to paraffin embedding. Tissue sections, cut to 5 μm, were stained with H&E or CD41 antibodie (Proteintech, IL, USA) and photographed. Three randomly selected fields per femur and spleen were imaged, and MKs were quantified.

#### Carotid artery thrombosis test

Ten days post-treatment, thrombosis was triggered by placing a 1 mm × 3 mm filter paper saturated with 10% FeCl_3_ onto left carotid artery. The artery was flushed with PBS and continuously tracked using a Transonic Model TS420 flowmeter (Transonic Systems, Ithaca, NY, USA).

#### Platelet aggregation

Platelet-rich plasma (PRP) was obtained from inferior vena cava. Platelet aggregation was detected using turbidimetric aggregation monitoring device (Helena Laboratories, Beaumont, TX, USA) after stimulation with ADP or collagen (Helena Laboratories, USA).

#### Tail bleeding assay

The distal tail was amputated and put in 37°C normal saline. Bleeding time was recorded as the time required for complete hemostasis.

#### Platelet activation

Mouse blood was collected in an anticoagulant tube containing 3.8% sodium citrate (1:9 v/v) and centrifuged at 100 × g for 10 min to obtain PRP. PRP was then centrifuged at 400 × g for 10 min to pellet platelets, which were washed and resuspended in modified Tyrode’s buffer. After resting for 1-2 hours at 25°C, platelets were activated with 10 μM ADP for 10 min at 37°C. Activated or resting platelets were incubated with PE-CD62P antibody (BioLegend, San Diego, CA, USA) for 20 min in the dark, followed by flow cytometry analysis.

#### Flow cytometric analysis *in vivo*

Ten days post-treatment, BM cells and spleens were incubated with RBC lysis buffer (Beijing 4A Biotech, Beijing, China) to remove red blood cells (RBCs). Samples were incubated with the following antibodies: PE-CD117 (c-Kit, Thermo Fisher Scientific, Waltham, MA, USA), FITC-CD41 (Elabscience, Wuhan, China), APC-CD42d (Thermo Fisher Scientific, Waltham, MA, USA), APC-61 (Thermo Fisher Scientific, Waltham, MA, USA), or PI (Beyotime, Shanghai, China). Then samples were detected by flow cytometer (Beckman Coulter, California, Brea, USA).

#### RNA sequencing

K562 cells were exposed to miltefosine at concentration of 40 μM for 3 days, followed by RNA extraction. RNA library was constructed and sequenced by Shanghai Majorbio Bio-pharm Biotechnology Co., Ltd. (Shanghai, China). Raw reads were quality-trimmed and filtered with SeqPrep and Sickle. All the sequencing data are available in NCBI Sequence Read Archive (SRA) under the accession number PRJNA1152712. DEGs were determined by applying a threshold of *p* < 0.05 and a fold change greater than 1.5. Then enrichment analysis of DEGs was conducted for DO, GO, KEGG, and Reactome pathways, respectively. Protein-protein interaction (PPI) networks were performed and visualized by String and NetworkX, respectively. Transcription factors were predicted from DEGs using the JASPAR database.

#### Molecular docking simulation

Structure of CCR5 (PDB ID: 4MBS) was retrieved from Protein Data Bank and UniProt database. Two-dimensional structure of miltefosine was obtained from PubChem database. AutoDock Vina was used to determine the binding ability between CCR5 and miltefosine. An affinity of ≤ −5.0 kcal/mol was considered indicative of a strong interaction, with lower values suggesting increased stability of the receptor-ligand complex.

#### Western blot

Total protein was isolated from K562 cells or BM cells of mice by RIPA lysis buffer (Beyotime, Shanghai, China) and analyzed via sodium dodecyl sulfate-polyacrylamide gel electrophoresis (SDS-PAGE). Separated proteins were transferred to polyvinylidene fluoride (PVDF) membranes, followed by overnight incubation with primary antibodies at 4°C. After a 1-hour incubation at 37°C with HRP-conjugated secondary antibodies, bands were detected using ECL reagent (Thermo Fisher Scientific, Waltham, MA, USA). Primary antibodies included: CCR5 (Abmart, TA6339), RAS (CST, 67648S), p-MEK (CST, 9154T), MEK (CST, 4694S), p-ERK (Proteintech, 28733-1-AP), ERK (Proteintech, 66192-1-Ig), p-JAK2 (Abmart, 56570), JAK2 (Abmart,TA6022), p-STAT3 (Proteintech, 28945-1-AP), STAT3 (Proteintech, 10253-2-AP), GATA1 (Abcam, ab308028), EGR1 (Abmart, T57177M), TAL1 (CST, 12831S), c-Myb (Proteintech, 17800-1-AP), and GAPDH (Abcam, ab8245).

#### DARTS assay

K562 cells were treated with RIPA lysis buffer (Beyotime, Shanghai, China) to isolate total protein. Protein samples were pre-treated with miltefosine (100, 200 or 400 μM) or DMSO for 1 h. Subsequently, samples were incubated with various dilutions of pronase E (1:400, 1:800, or 1:1600) at 40°C for 10 min. CCR5 expression was assessed via western blot analysis.

#### SPR assay

The activator solution was prepared by mixing 400 mM 1-Ethyl-3-(3-dimethylaminopropyl)carbodiimide and 100 mM N-Hydroxysuccinimide immediately before injection. The CM5 sensor chip was activated with this mixture at a flow rate of 10 μL/min for 420 s. For ligand immobilization, CCR5 protein (KACTUS Biosystems, Shanghai, China) was diluted to 45 μg/mL in immobilization buffer and injected into the sample channel (Fc2) at 10 μL/min, achieving typical immobilization levels of 3700 RU, while the reference channel (Fc1) did not undergo ligand immobilization. The chip was deactivated by injecting 1 M ethanolamine hydrochloride at 10 μL/min for 420 s. For analyte binding, miltefosine was diluted in analyte buffer to six concentrations (12.5, 6.25, 3.125, 1.5625, 0.78, and 0 μM) and injected into the sensor channels (Fc1-Fc2) at 10 μL/min, with a 120 s association phase followed by a 200 s dissociation phase. The association and dissociation steps were conducted in analyte buffer. This process was repeated for six cycles in ascending order of miltefosine concentration, with each cycle followed by natural dissociation.

#### Immunofluorescence assay

Cells were transferred onto slides, incubate with 4 % paraformaldehyde, and treated with 0.05 % Triton X-100. After staining with primary antibodies and secondary antibodies, samples were counterstained with DAPI and analyzed using fluorescence microscopy (Leica, Germany). Primary antibodies included: CCR5 (Abmart, TA6339), GATA1 (Abcam, ab308028), EGR1 (Abmart, T57177M), β-tubulin (Abcam, ab6046).

### Quantification and statistical analysis

Data are presented as means ± SD from a minimum of three independent experiments.Statistics were performed using Graph Pad Prism 8 Software. Statistical significance between two groups was determined using two-tailed Student’s t-tests. For comparisons among multiple groups, one-way ANOVA and two-way ANOVA followed by Tukey’s post hoc test was utilized. Differences were considered statistically significant at *p* < 0.05.
