# Supplementary material for: Targeting CCR5 with miltefosine as a therapeutic strategy for thrombocytopenia
Source: iScience. 2025 Apr 8;28(5):112379. doi: 10.1016/j.isci.2025.112379 (PMC12032913; doi:10.1016/j.isci.2025.112379)
Supplement: Document S1. Figures S1–S9 [file mmc1.pdf]

iScience, Volume *28*

Supplemental information

Targeting CCR5 with miltefosine

as a therapeutic strategy for thrombocytopenia

Qinyao Li, Ting Zhang, Zhichao Li, Xiao Qi, Xinyue Mei, Sheng Liu, Siyu He, Gan Qiao, Rong Li, Hongping Shen, Jing Zeng, Feihong Huang, Shuang Dai, Sirui Li, Jiesi Luo, Jianming Wu, and Long Wang

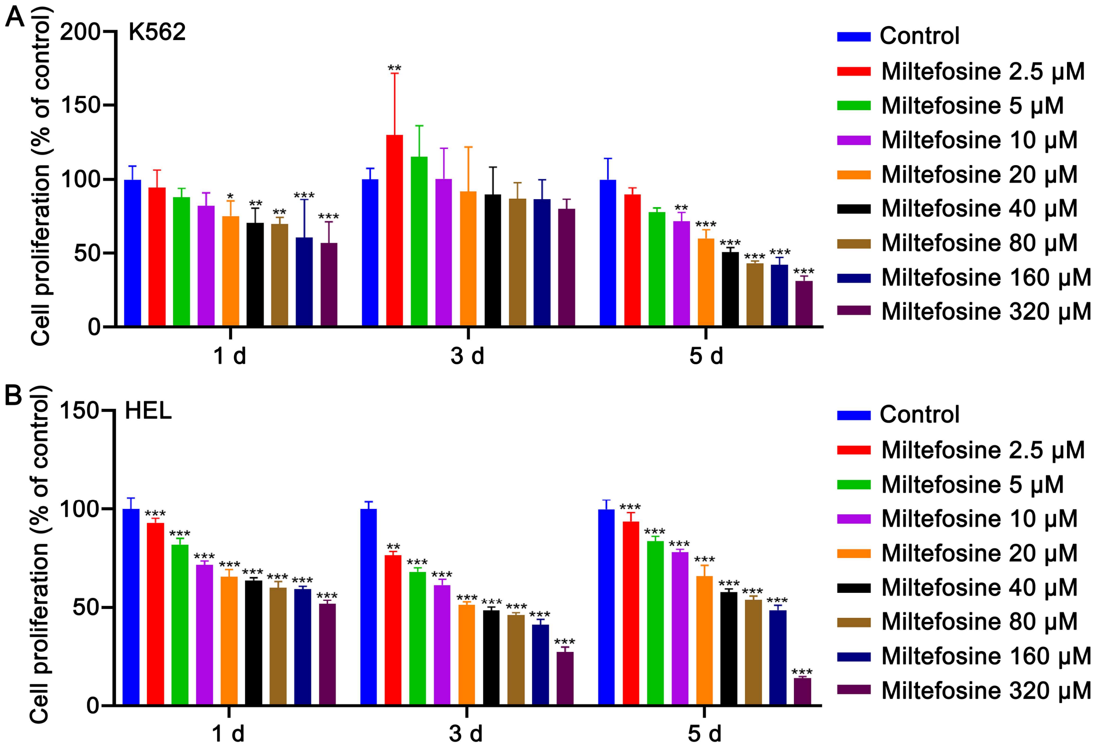

Figure S1. CCK-8 assay detection the effect of miltefosine on cell proliferation, related to Figure 1.

(A) Proliferation rates of K562 cells treated with miltefosine across a concentration range of 2.5 to 320 μM over 1, 3, and 5 days.n=3.

(B) Proliferation rates of HEL cells following treatment with miltefosine at concentrations ranging from 2.5 to 320 μM over 1, 3, and 5 days. n=3.

Statistical significance in (A-B) was calculated using a one-way ANOVA test. *$p < 0.05$, **$p < 0.01$, ***$p < 0.001$, vs control group .Error bars represent mean ± SD.

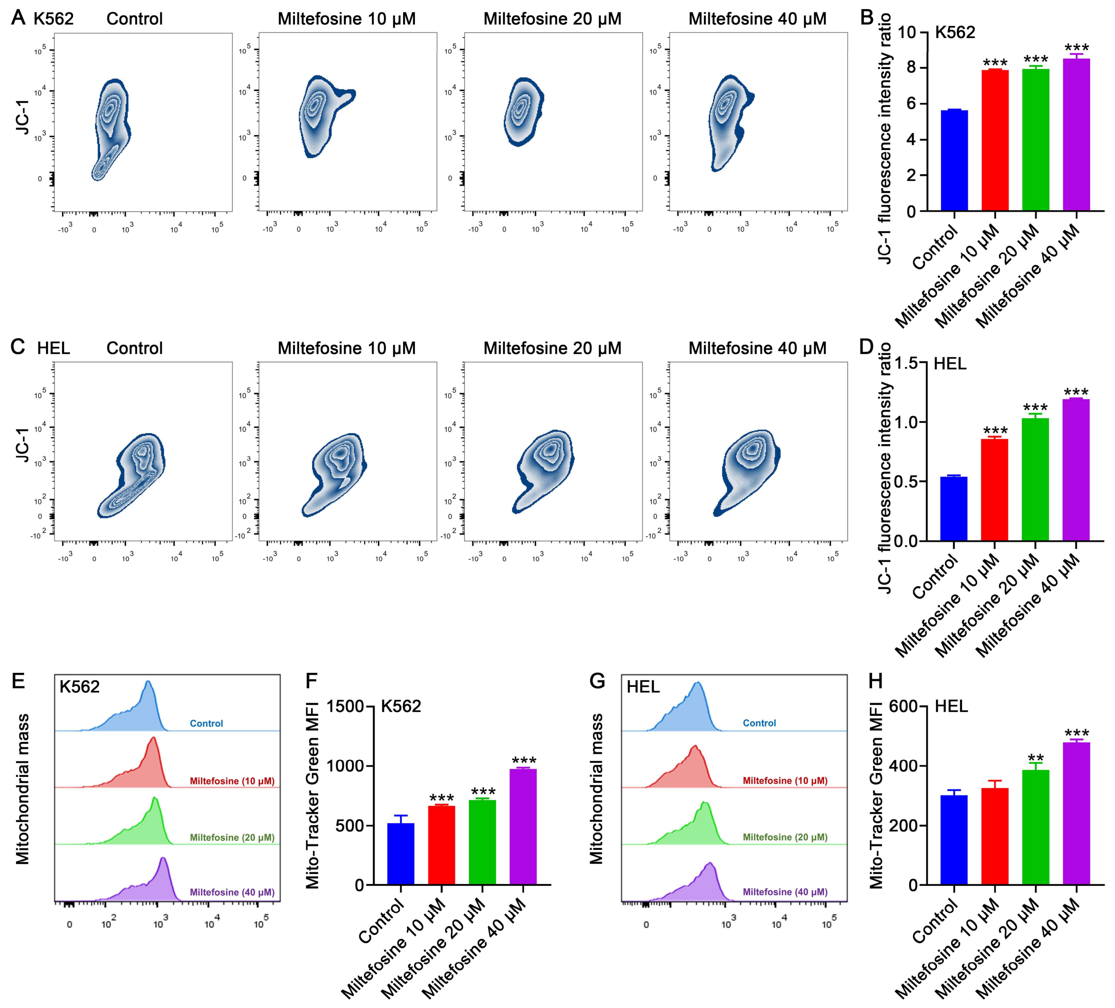

Figure S2. The effects of miltefosine on mitochondria, related to Figure 2.

(A, B) Flow cytometric analyses and quantification of mitochondrial membrane potential (MMP) in K562 cells treated with miltefosine (10, 20, and 40 μM) for 5 days, using JC-1 staining. n=3.

(C, D) Flow cytometric analyses and quantification of MMP in HEL cells from each treatment group, using JC-1 staining. n=3.

(E, F) Flow cytometric analyses and quantification of mitochondrial mass in K562 cells, assessed by Mito-Tracker Green staining. n=3.

(G, H) Flow cytometric analyses and quantification of mitochondrial mass in HEL cells from each treatment group. n=3.

Statistical significance in (B,D,F,H) was calculated using a one-way ANOVA test. ***p* < 0.01, ****p* < 0.001, vs control group.Error bars represent mean ± SD.

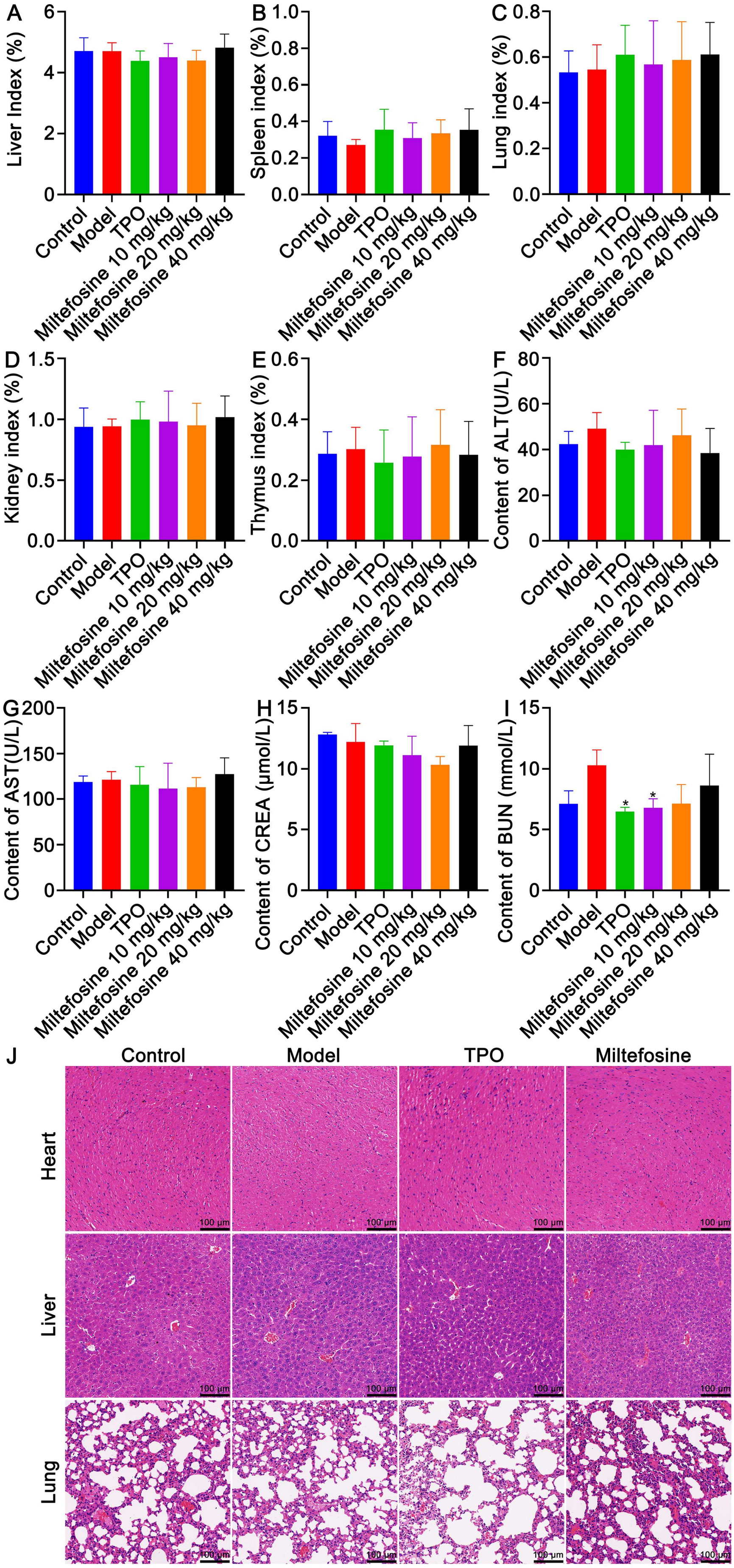

A
Liver Index (%)
B
Spleen index (%)
C
Lung index (%)
D
Kidney index (%)
E
Thymus index (%)
F
Content of ALT(U/L)
G
Content of AST(U/L)
H
Content of CREA (μmol/L)
I
Content of BUN (mmol/L)
Control
Model
TPO
Miltefosine 10 mg/kg
Miltefosine 20 mg/kg
Miltefosine 40 mg/kg
J
Control
Model
TPO
Miltefosine
Heart
Liver
Lung
100 μm

Figure S3. Safety evaluation of miltefosine in radiation-induced thrombocytopenia mice, related to Figure 3.

(A-E) Organ indices of liver, spleen, lung, kidney, and thymus from control, model, TPO (3000 U/kg), and miltefosine (40 mg/kg)-treated groups on day 16.n=6.

(F-I) Serum biochemical measurement of alanine aminotransferase (ALT), aspartate aminotransferase (AST), creatinine (CREA), and blood urea nitrogen (BUN) from control, model, TPO (3000 U/kg), and miltefosine (40 mg/kg)-treated groups on day 16.n=3.

(J) Hematoxylin and eosin (H&E) staining of heart, liver and lung from each group. Scale bar: 100 μm.

Statistical significance in (A-I) was calculated using a one-way ANOVA test. *$p < 0.05$,vs model group . Error bars represent mean ± SD.

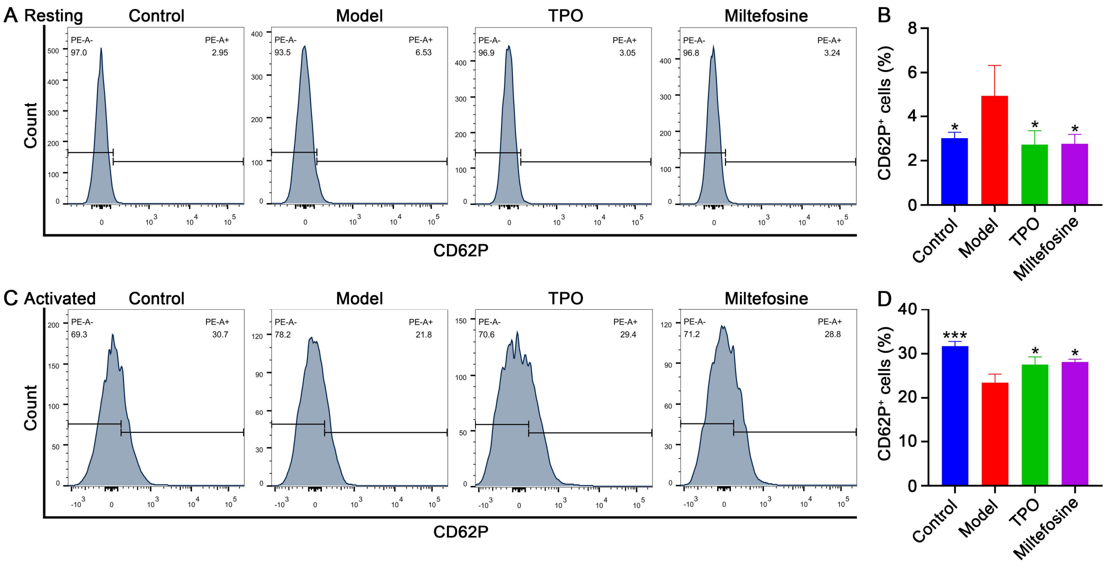

Figure S4. The effects of miltefosine on platelet activation, related to Figure 3.

(A, B) Flow cytometric analyses and quantification of CD62P⁺ cells on platelets in control, model, TPO (3000 U/kg), and miltefosine (40 mg/kg)-treated groups.n=3.

(C, D) Flow cytometric analyses and quantification of CD62P⁺ cells on platelets after ADP stimulation in each group.n=3.

Statistical significance in (B,D) was calculated using a one-way ANOVA test. *p < 0.05, ***p < 0.001, vs model group . Error bars represent mean ± SD.

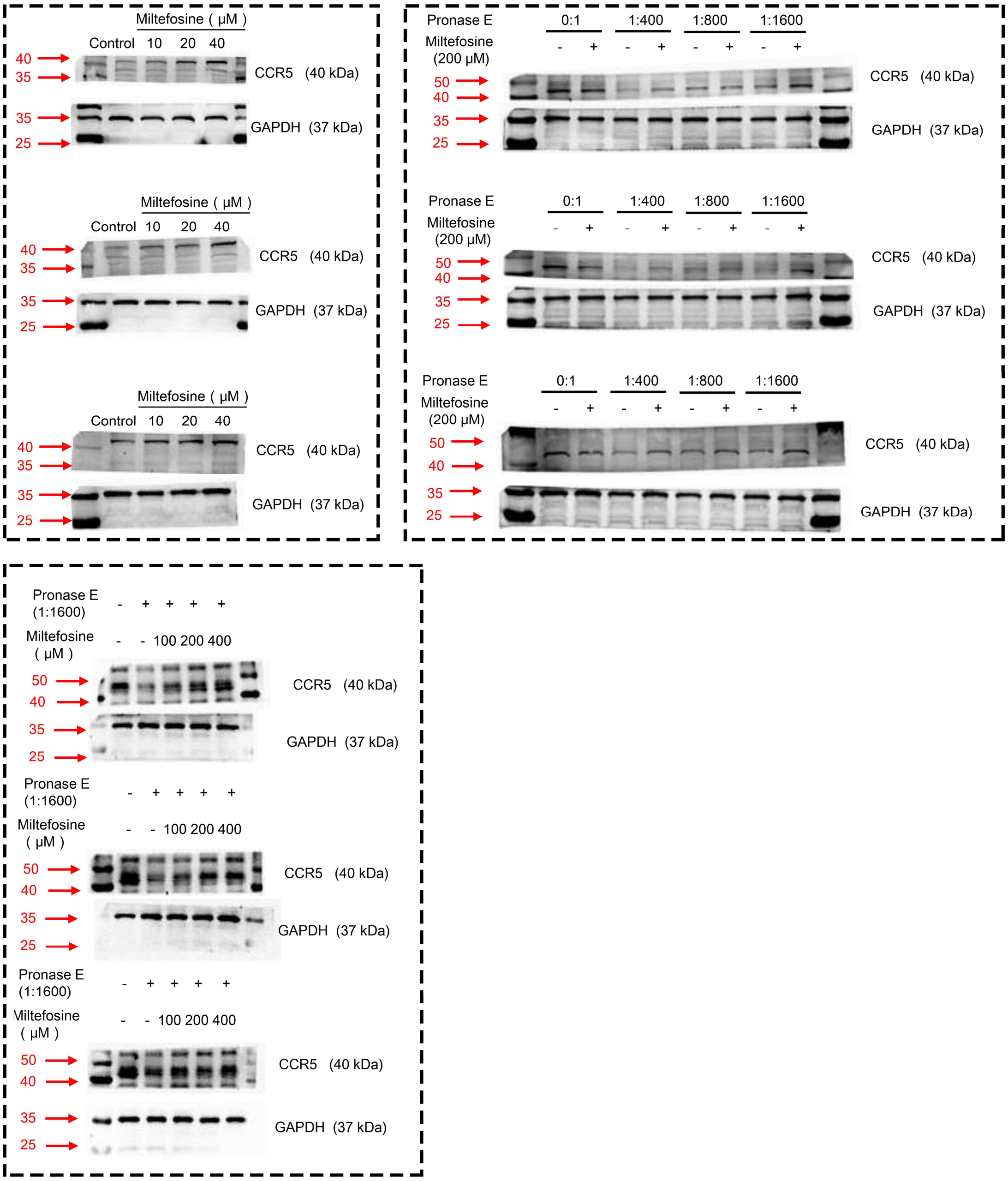

Figure S5. Source data of western blot analysis, related to Figure 6.

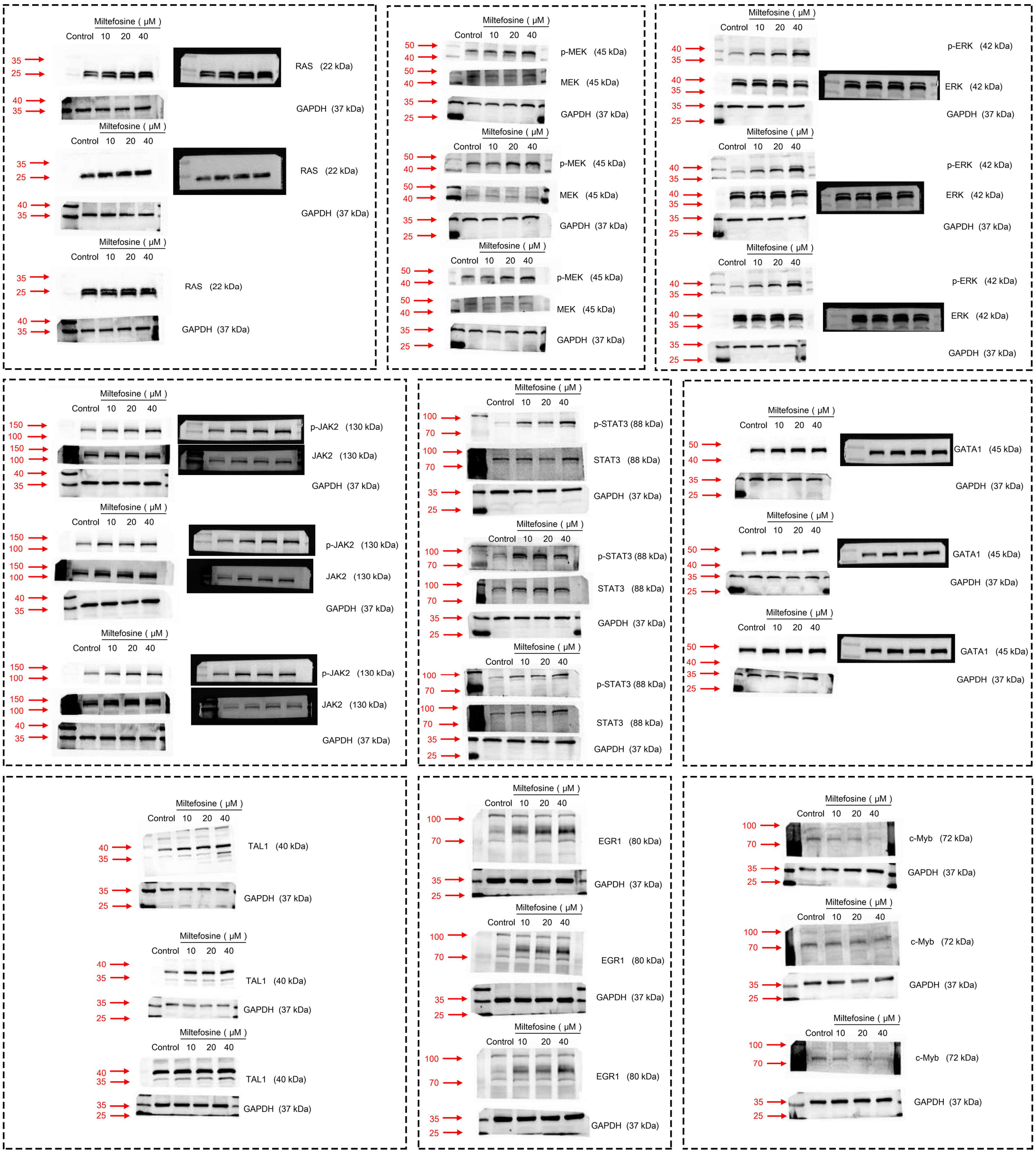

Figure S6. Source data of western blot analysis, related to Figure 7.

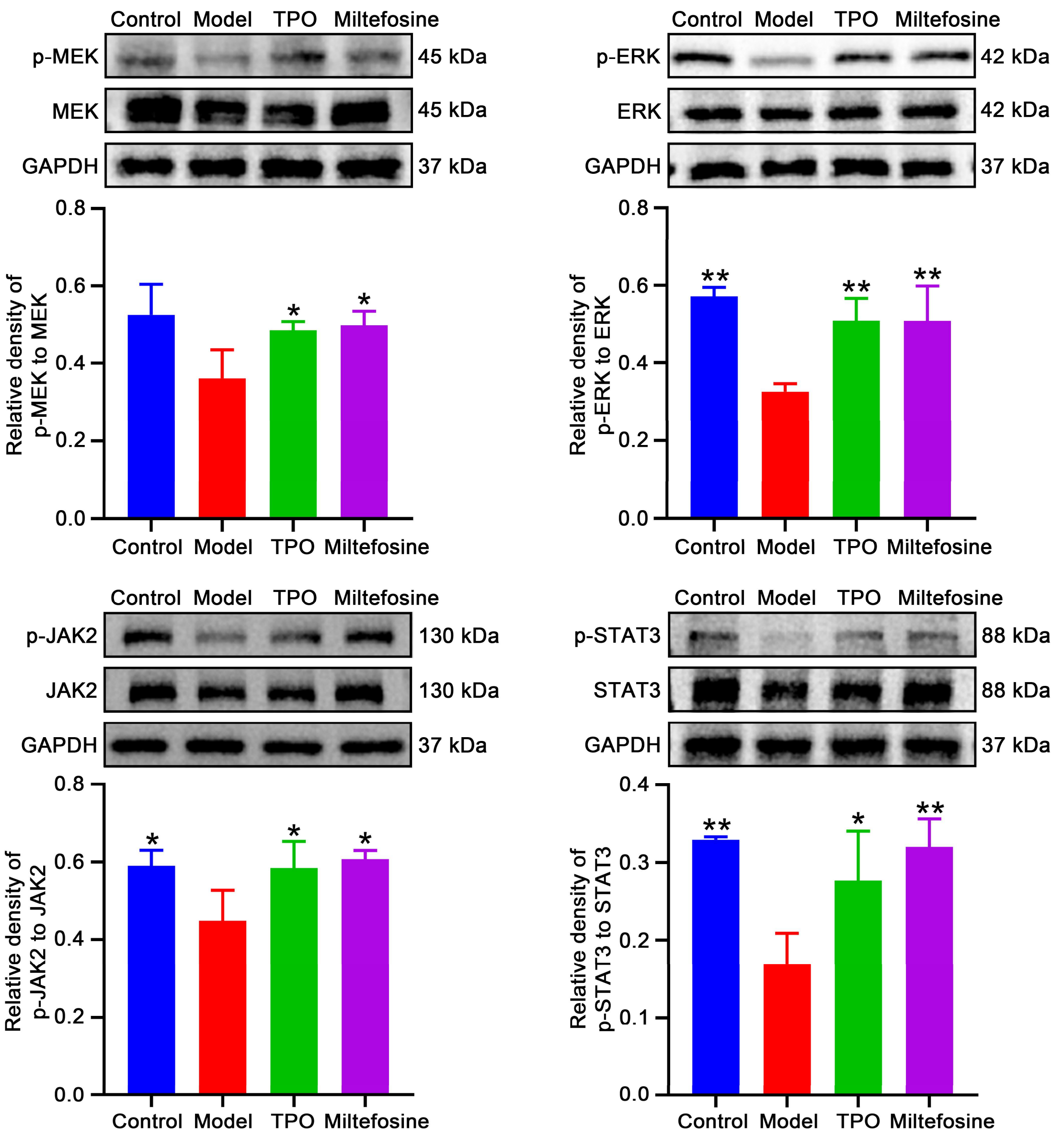

Figure S7. The effects of miltefosine on the phosphorylation levels of MEK, ERK, JAK2 and STAT3 in bone marrow cells of radiation-induced thrombocytopenia mice , related to Figure 7.

Statistical significance was calculated using a one-way ANOVA test.n=3.*$p < 0.05$, **$p < 0.01$, vs model group . Error bars represent mean ± SD.

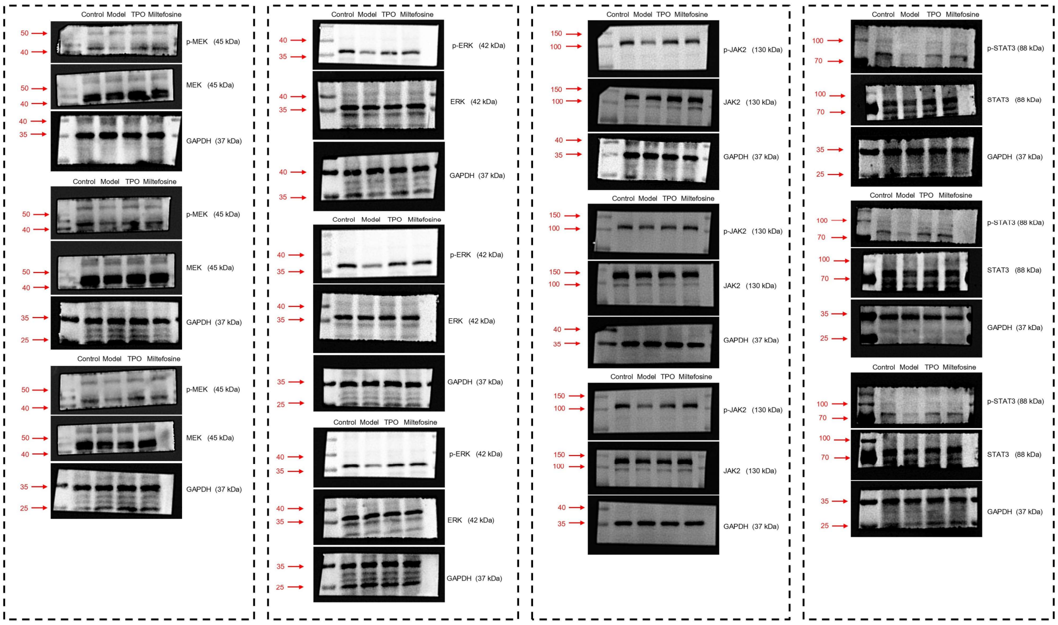

Figure S8. Source data of western blot analysis, related to Figure S7.

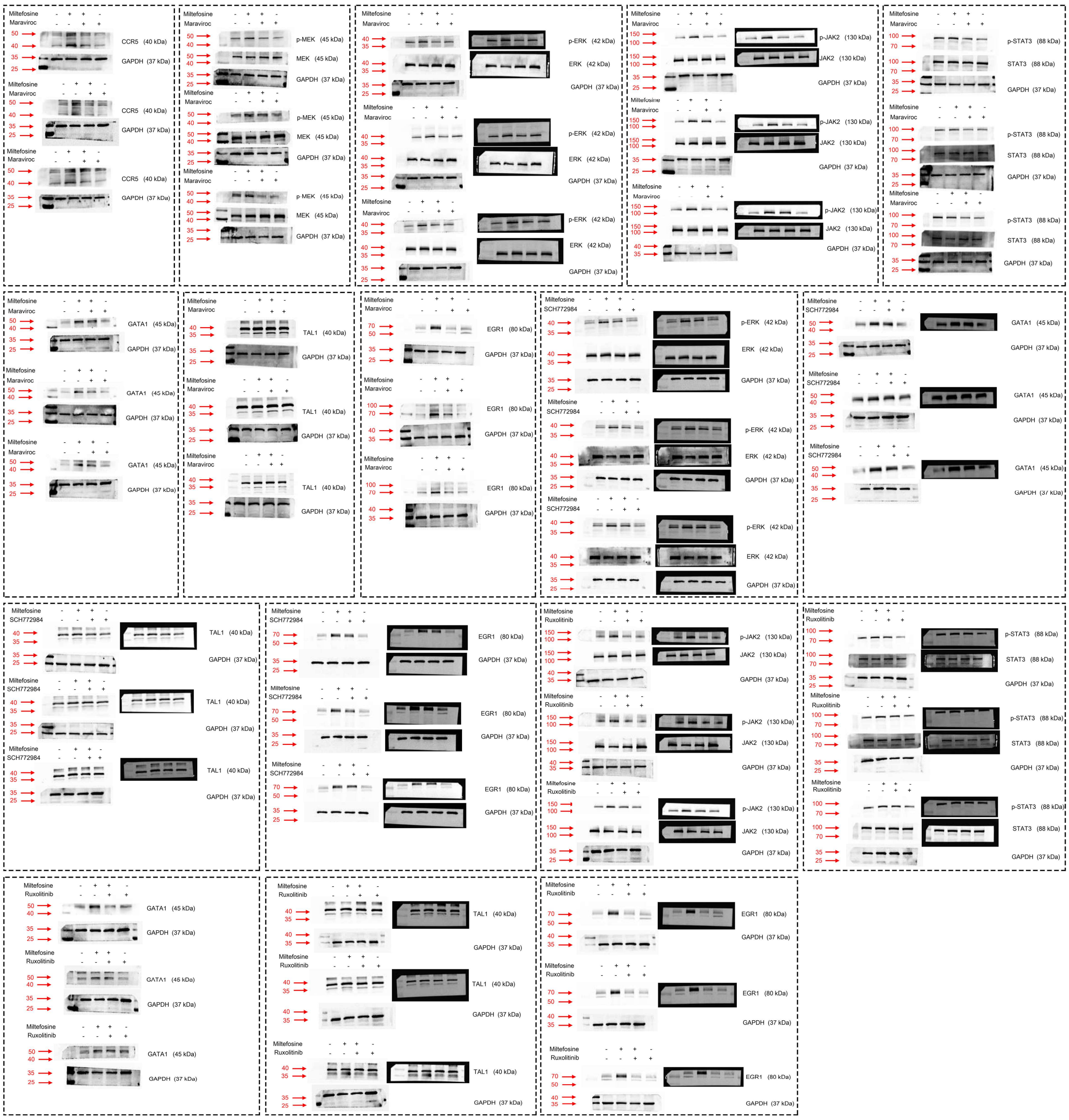

Figure S9. Source data of western blot analysis, related to Figure 9.
